# circCYP24A1 facilitates esophageal squamous cell carcinoma progression through binding PKM2 to regulate NF-κB-induced CCL5 secretion

**DOI:** 10.1186/s12943-022-01686-7

**Published:** 2022-12-13

**Authors:** Lina Gu, Yang Sang, Xixi Nan, Yang Zheng, Fei Liu, Lingjiao Meng, Meixiang Sang, Baoen Shan

**Affiliations:** 1 Research Center, the Fourth Hospital of Hebei Medical University, 050017 Shijiazhuang, Hebei People’s Republic of China; 2 Animal Center, the Fourth Hospital of Hebei Medical University, Shijiazhuang, Hebei People’s Republic of China; 3 Tumor Research Institute, the Fourth Hospital of Hebei Medical University, 050017 Shijiazhuang, Hebei People’s Republic of China

**Keywords:** Esophageal squamous cell carcinoma, circCYP24A1, PKM2, NF-κB, CCL5

## Abstract

**Background:**

Esophageal squamous cell carcinoma (ESCC) is a common gastrointestinal malignant tumor, while the molecular mechanisms have not been fully elucidated. Multiple circular RNAs have been reported to involve in the onset and progression of malignant tumors through various molecular mechanisms. However, the clinical significance and functional mechanism of most circRNAs involved in the progression of ESCC remains obscure.

**Methods:**

RNA-Seq was used to explore potential circRNAs in participated in 5 pairs of ESCC and their corresponding normal esophageal tissues. The up-regulated circCYP24A1 was selected. Fluorescence in situ hybridization was cunducted to verificated the expression and intracellular localization of circCYP24A1 by using the tissue microarray. The Kaplan–Meier method and Cox proportional hazards model was used to examine the potential prognostic value of circCYP24A1 on overall survival of ESCC patients. The biological function were confirmed by gain- and loss-of-function approaches in vivo. mRNA expression profile microarray was proformed to investigate the downstream signaling pathways involved in circCYP24A1. RNA pull-down assay and mass spectrometry were performed to identify the proteins associated with circCYP24A1. Rescue experiments were carried out to identified hypothetical regulatory role of circCYP24A1 on ESCC progression in vivo and in virto.

**Results:**

In this study, we identified circCYP24A1 in ESCC tissues by RNA sequencing, which is up-regulated in 114 cases of ESCC tissues and acts as a novel prognosis-related factor. Moreover, circCYP24A1 promoted the ability of proliferation, migration, invasion and clone formation in vitro, as well as tumor growth in vivo. Mechanistically, chemokine (C-Cmotif) ligand 5 (CCL5) is functional downstream mediator for circCYP24A1, which is screened by mRNA microarray. Moreover, circCYP24A1 physically interacts with M2 isoform of pyruvate kinase (PKM2). Rescue experiments showed that PKM2 knockdown partly reverses the promotional effects of circCYP24A1. It was revealed that circCYP24A1 increases secretion of CCL5 through the mechanism mainly by interacting with PKM2, an activator of NF-κB pathway, and thereby accelerate malignant progression of ESCC.

**Conclusions:**

Up-regulated circCYP24A1 could activate NF-κB pathway by binding PKM2, which promotes the secretion of CCL5 and accelerate malignant progression of ESCC. Our fndings recommended a novel function for circCYP24A1 as a potential effective biomarker for judging prognosis and a therapeutic target in ESCC.

**Supplementary Information:**

The online version contains supplementary material available at 10.1186/s12943-022-01686-7.

## Background

Esophageal carcinoma (EC) is the common malignant tumor of the digestive tract globally [1]. However, the burden from EC was more common in Asia, especially in China, than in any other countries [2]. The incidence characteristics of EC in China are different from those in other countries, most of which are esophageal squamous cell carcinoma (ESCC), showing certain familial clustering and poor prognosis [3]. Despite the advances in the diagnosis and treatment on ESCC, the prognosis of ESCC patients is still not good. This is mainly due to inapparent symptoms at early stage and prone to metastasis [1, 3]. Therefore, it is essential to investigate the molecular mechanism of ESCC, so as to better understand the progress and metastasis of ESCC, and to provide more effectively therapeutic targets and prognostic biomarkers for clinical diagnosis and treatment.

As one of the hallmarks of tumor cells, activating invasion and metastasis is a multistep complex process, involving the activation of various vital genes and signal pathways [4–6]. With a deeper understanding of non-coding RNA (ncRNA) biology, researchers have recognized that ncRNA is essential for tumorigenesis [7–9]. Circular RNAs (circRNAs) are a newly discovered class of covalently closed RNA molecules that do not contain the 5 ' cap and the 3' terminal poly (A) tail [10]. With the rapid development of high-throughput sequencing technology, multiple studies have confirmed that circRNA exists widely in transcriptome and has histological specificity [11, 12]. Due to the special structure, circRNAs cannot be hydrolyzed by exonuclease, which makes them more stable than linear RNAs [13]. Some high abundance circRNAs may be used as biomarkers for tumor diagnosis and treatment. At present, circRNAs have been found to regulate transcription in the following four ways. First, circRNAs act as competitive endogenous RNAs (ceRNAs) competitively bind microRNAs (miRNAs) competitively and regulate mRNA by targeting miRNAs [14]. Second, they promote or inhibit transcription by interacting with transcription factors [15]. Third, they can encode polypeptides or proteins [16]. Fourth, they act as a sponge or scaffold for proteins by binding to RNA binding proteins (RBPs) [17]. Recently, the cross-linked immunoprecipitation (CLIP) dataset showed that circRNAs play an improtant role in tumor progression by interacting with RBPs [12]. For example, circTHBS1 can bind to RBP HuR to enhance INHBA mRNA stability, thereby activating TGF-β pathway [18]. CircNSUN2 can combine with IGF2BP2 and HMGA2 to form a ternary complex, which could promote HMGA2 mRNA stabilization [19]. Hu, et al. found that circGSK3β interacts with GSK3β directly and inhibits the activity of GSK3-β, promoting migration and invasion of ESCC [6]. However, the clinical significance and functional mechanism of most circRNAs involved in the progression of ESCC remains obscure.

In this study, we identified hsa_circ_0060927 (originate from the back splicing of cytochrome P450 family 24 subfamily A member 1, also known as circCYP24A1) in ESCC tissues by RNA sequencing, which is up-regulated in ESCC tissues and acts as a novel prognosis-related factor in ESCC. Mechanistically, circCYP24A1 enhanced ESCC cell proliferation, colony formation, migration and invasion, which was partly by stimulating the secretion of chemokine (C-Cmotif) ligand 5 (CCL5), a downstream target gene of the NF-κB pathway. Moreover, it was verified that circCYP24A1 could promote the phosphorylation of NF-κB and facilitates ESCC progression by interacting with M2 isoform of pyruvate kinase ( PKM2). Therefore, the study revealed that up-regulated circCYP24A1 could activate NF-κB pathway by binding PKM2, which promotes the secretion of CCL5 and accelerate malignant progression of ESCC. Our fndings recommended a novel function for circCYP24A1 as a potential effective biomarker for judging prognosis and a therapeutic target in ESCC.

## Materials and methods

### RNA sequencing ( RNA-Seq), identification of human circRNAs

This study has been approved by the Medical Ethics Committee of the Fourth Affiliated Hospital of Hebei Medical University. CircRNA expression profiles of ESCC (*n* = 5) and their corresponding normal esophageal tissues (*n* = 5) were compared by using RNA-Seq. Clinical information of the ESCC patients is summarized in Table S1. Total RNA from each sample was isolated using TRIzol reagent (Invitrogen, USA). After confirming the integrity and purity of RNA, the Ribo-Zero rRNA Removal Kit (Illumina, San Diego, CA, USA) and RNase R (Epicentre, USA) were uesd to remove the rRNA and linear RNA, respectively. Finally, the circular RNA was enriched. RNA-seq libraries were prepared with pretreated RNAs and subjected to deep sequencing on an Illumina HiSeq™ 4000 Sequencer at Wankangyuan (Tianjin) Gene Technology Co., Ltd. The reads were adjusted to the reference genome/transcriptome with STAR software, and circRNAs were identified using find_circ and CIRI software.

### Cell culture, drug treatment and transfection

Four kinds of human esophageal cancer cells inculding TE1, KYSE30, KYSE150 and KYSE170 were chosen to study, which were purchased from Procell Life Science&Technology Co., Ltd. (Wuhan, Hubei, China). There are no mycoplasma contamination among them. All cell lines were culture with RPMI1640 medium (GIBCO, USA) containing with 10% fetal bovine serum (FBS), 100 U/ml of penicillin and 100 μg/ml streptomycin, which were maintained at 37 °C in a humidified atmosphere of 5% CO_2_. For drug treatment, cells were cultured with or without 10 ng/mL recombinant human RANTES (CCL5) (Peprotech, USA) in medium. For transfection, siRNAs were supplied by Guangzhou RiboBio Co.,Lid.( Guangzhou, China). The hsa_circ_0060927 sequence was cloned into the pLC5-ciR vector and synthesized by Geneseed Biotech Co.( Guangzhou, China). According to the manufacturer’s instruction, ESCC cells inoculated in 6-well cell plates (1 × 10^6^) were transfected with pLC5-ciR vector or siRNA by using FuGENE HD transfection Reagent (Promega, USA) or HiperFect transfection Reagent (Promega, USA). 6 h later, the complete medium was used to instead of the transfection medium. After a further culture for 48 h, the cells were collected.

### Patients and specimens

In this study, 114 ESCC tissues and 66 normal esophageal tissues derived from HEsoSqu180Sur08 tissue microarray were purchased from Shanghai Outdo Biotech Company. The clinicopathological characteristics and survival status of ESCC patients can be obtained from the follow-up data. All the above samples were obtained after getting informed consents of patients and their families. Ethics Committee of Shanghai Outdo Biotech Company had approved this study.

### Genomic DNA (gDNA) /RNA extraction and RNase R treatment

The gDNA and total RNA from ESCC cells and malignant tumor tissues was extracted using the gDNA extraction kit (TANGEN, China) and TRIzol reagent (Invitrogen, USA), respectively. Extracted RNAs were treated with RNase R (Epicenter, 3U/mg 37 °C, 15 min).

### cDNA synthesis, and quantitative real-time PCR assays (qRT-PCR) /RT-PCR

The GoScript Reverse Transcription System (Promega, USA) was used to synthesize cDNA. The amplification process was performed using GoTaq qPCR Master Mix (Promega, USA) and GoTaq Green Master Mix. The primers in Table S2 were used for PCR amplification. U6 nsRNA and GAPDH were used as internal controls for circRNAs and mRNA, respectively. Using the 2^−△△CT^ method, the fold changes of the target genes were calculated. The RT-PCR products were analyzed with 2% agarose gel electrophoresis, and DNA fragmentation visualized using Safe Green (Monad, China).

### Actinomycin D assay

Cells were inoculated in 6-well plates before actinomycin D (AdooQ Bioscience, USA) treatment. All cells were treated with 2 μg/mL actinomycin D for 4 h, 8 h, 12 h and 24 h, whereas the control group was without any treatment. Then, RNA was extracted and subjected to qPCR detection of RNA stability.

### Fluorescence in situ hybridization(FISH)

FISH was conducted in ESCC cells and neoplastic tissues with biotin-labeled circCYP24A1 probe (Guangzhou Geneseed Biotech Co., Ltd., China). The primers in Table S2 were used for FISH assay. In ESCC cells, KYSE30 and KYSE150 cells were inoculated on the glass cover slips.On the next day, the cells were fixed at room temperature with 4% paraformaldehyde and prehybried for 1 h. Then, the cells were were overlaid with circCYP24A1 probe overnight at 37 °C. In neoplastic tissues, the ESCC tissue microarray slides were dewaxed in xylene and gradient ethanol hydration. Pre hybridization and hybridization process were the same as those of cell FISH. The fluorescence signal of circCYP24A1 was detected using Cy5-Streptavidin Conjugate (ZyMAXTM Grade, Invitrogen). Nuclei were counterstained with DAPI for 10 min. The images were captured with the confocal microscope (LSM 900, Zeiss, Germany).

### Cell proliferation and colony formation assays

For cell proliferation assays, ESCC cells were stored in 96-well plates with the density of 5 × 10^3^ cells/well for overnight. Cell Counting Kit-8 (MedChemExpress, USA) kit was used to measure cell proliferation at 24, 48, 72, and 96 h according to the manufacturer’s instructions. For colony formation assays, ESCC cells were stored in 6-well plates with the density of 1000 cells per well. After culturing for 10 days, the colonies were fixed with 4% paraformaldehyde for 20 min and stained with crystal violet for 10 min. Cell colonies were photographed and analyzed.

### Wound healing assay

Cells treating with vectors or siRNA were inoculated in 6-well plates. After 24 h of incubation, two vertical bottom lines were drawn in the cells with a 200 μL gunhead. The scratched areas were photographed at 0 and 24 h after scratching using a microscope (DFC295, Leica, Buffalo Grove, United States). Scratch healing rate (%) = [(0 h scratch width-24 h scratch width)/ 0 h scratch width]*100%.

### Transwell assays and inverted invasion assays

For invasion assay, Matrigel (BD biosciences, USA) was evenly spread in the chamber at 37 ℃ overnight. Invasion assays were performed using Transwell chambers (BD biosciences, USA) without Matrigel. Cells were inoculated in the upper chamber at a density of 4 × 10^4^ cells/chamber for 24–48 h. After crystal violet staining, 3 visual fields were randomly observed and photographed under an inverted phase contrast microscope (DFC295, Leica, Buffalo Grove, United States).

### Inverted invasion assays

The detailed steps of this experiment were mainly refered to Chen's previous article [19]. Briefly, Matrigel (BD biosciences, USA) was evenly spread in the chamber at 37 ℃ for 1-2h. The chambers were put upside down and cells were inoculated in the outer chamber surface at a density of 4 × 10^5^ cells/chamber for 5h. The chambers were inverted again and placed in the medium without fetal calf serum. 500 μl medium containing 10% FBS and 150ng/ml IGF (Sino Biological, China) were added into the chambers. After incubation with 4nM Calcein (Invitrogen, Carlsbad, USA), the images were captured with the confocal microscope (LSM 900, Zeiss, Germany) at 10 μm intervals. ImageJ was used to assess the fluorescence intensity of each section. The relative invasiveness was judged by calculating the ratio of the sum of the intensities of all sections over 30 µ m to the sum of the total intensities of all sections.

### Microarray analysis

The total RNAs from control or si-circCYP24A1 KYSE150 cells were quantified by NanoDrop ND-2000 (Thermo Scientific). RNA integrity was detected by Agilent Bioanalyzer 2100 (Agilent Technologies). After the RNA quality inspection is qualified, the sample labeling, hybridization and elution of the chip were performed based on the chip standard protocols. Briefly, the total RNA was reverse transcribed into double stranded cDNA, and the cDNA labeled with Cyanine-3-CTP (Cy3)was further synthesized.The labeled cDNA was hybridized with Agilent SurePrint G3 Human Gene Expression v3 Microarray (8 × 60 K). After elution, the arrays were scanned by Agilent Scanner G2505C (Agilent Technologies) and analyzed by Feature Extraciton software (version10.7.1.1, Agilent Technologies) and Genespring software (version13.1, Agilent Technologies). Differential gene screening was performed by T test using the p value and fold change value. The screening criteria were the fold change value of up-regulation or down-regulation < 2 and the *p* value < 0.05.

### Enzyme-linked-immunosorbent serologic assay (ELISA)

Cell culture supernatants were collected and analyzed for cytokines production using Human CCL5/RANTES ELISA kit (MULTI SCIENCES, China) according to the manufacturer’s instructions.

### RNA FISH-immunofluorescence microscopy.

To detect co-localization of circCYP24A1 with PKM2 proteins, ESCC cells were inoculated on the glass cover slips.On the next day, the cells were fixed at room temperature with 4% paraformaldehyde and prehybried for 1 h. Then, the cells were were overlaid with biotin-labeled circCYP24A1 probe probe overnight at 37 °C. The cells were blocked by 10% BSA for 30 min at 37 °C. Subsequently, the ESCC cells were incubated with PKM2 antibody (1:200) at room temperature for 1 h and Alexa Fluor™ 488-conjugated secondary antibodies (1:200) and Cy5-Streptavidin Conjugate (ZyMAXTM Grade, Invitrogen) for 1 h at 37 °C. Nuclei were counterstained with DAPI for 10 min. The images were captured with the confocal microscope (LSM 900,Zeiss, Germany).

### RNA pull-down assays

The ChIRP probe against circCYP24A1 backsplice junction region was synthesized by RiboBio (Guangzhou, China). The primers in Table S2 were used for RNA pull-down assays. In brief, 1 × 10^7^ cells were incubated with 3ug of biotin-labeled negative or circRNA probe for 2 h and treated with 50 μl of Streptavidin C1 magnetic beads (Invitrogen) for another hour. The beads were washed with wash buffer for three times. The eluted proteins were identified by mass spectrometry or detected by western blot analysis.

### Western blot analysis

ESCC cells were lysed with RIPA protein lysate or IP lysis buffer, which stayed on ice for 30 min, centrifuged and taken supernatant. Proteins quantitated by BCA were analyzed with 10% SDS-PAGE. PVDF membranes (Millipore) were uesd to transfer the electrophoretic separated proteins, and were sealed by blocking liquid for 1 h and incubated with antibodies against PKM2 (1:1000 dilution; #4053, Cell signaling, Danvers, MA, USA), NF-κB p65 (1:1000 dilution; #8242, Cell signaling, Danvers, MA, USA), p-NF-κB p65 (Ser536; 1:1000 dilution; #3033 Cell Signaling Technology, Danvers, MA, USA) and β-actin (1:1000 dilution; ab8227, Abcam, USA) overnight at 4 °C. Electrochemiluminescence (ECL) detection kit (Thermo Scientific, USA) were used to measure the bands. Image J software was used to analyze the gray value of protein bands.

### In vivo tumorigenesis assays

The in vivo experiment was approved by the Institutional Animal Care and Use Committee (IACUC) of the Fourth Affiliated Hospital of Hebei Medical University (IACUC approval No.2022122). All of the NOD-SCID mice were obtained from SPF (Beijing) Biotechnology Co., Ltd. For the in vivo tumorigenesis assays, NOD-SCID mice were randomly divided into four groups and were subcutaneously injected with 1 × 10^7^ KYSE170 infected with pLC5-ciR vector or circCYP24A1 cells in 200µL serum-free 1640. On the ninth day, the mice were were injected with cholesterol-modified PKM2 or NC siRNA (5 nmol/ 20 g, RioBio, Guangzhou, China) intratumorally. The injection frequency was once every 3 days, six times in total. The tumor volume was measured and recorded at each injection. Subsequently, the mice were sacrificed and dissected. One part was used for qRT-PCR detection, the other part was used f to be performed immunohistochemistry (IHC) assay.

### Statistical analysis

SPSS22.0 software (SPSS Inc, Chicago, IL, USA) was used for statistical analysis in this research. Mean ± S.D. was used to represente the results of qRT-PCR and ELISA. The means between different groups were examined by student’s t-test. Chi-square test was used to analysis the methylation statuses of genes among different groups. The Kaplan–Meier method was used to draw survival curves. The univariate analysis and mltivariate analysis was used to examine the possible prognostic role of different elements. *P* < 0.05 or *P* < 0.01 was indicated that the difference was statistically significant.

## Results

### Characteristics of circCYP24A1 in ESCC

**Fig. 1:**
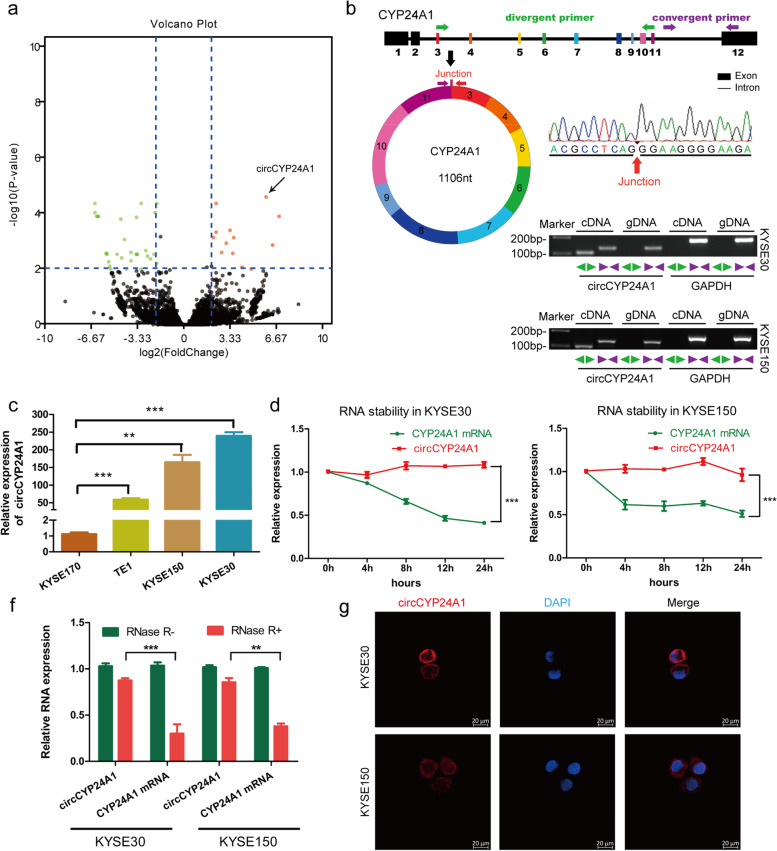
Characteristics of circCYP24A1 in ESCC. **a** A volcano plot shows 10,633 differentially expressed circRNAs in five paired samples of ESCC by RNA-Seq. Cut off is Log 2 (Fold Change) > 1.0 or <  − 1.0, P value < 0.05. **b** Upper panel: The genomic structure and backsplice junction site of circCYP24A1 was analyzed by circBase database and Sanger sequencing. Lower panel: The expression of circCYP24A1 in KYSE30 and KYSE150 cells was detected by RT-PCR. **c** The expression of circCYP24A1 in ESCC cells was detected by qRT-PCR. **d** RNA abundance of circCYP24A1 and CYP24A1 treated with Actinomycin D (2 μg/ml) was detected by qRT-PCR. ***P < 0.001. **f** Resistance of circCYP24A1 and CYP24A1 to digestion with RNase R exonuclease was detected by qRT-PCR. **P < 0.01, ***P < 0.001. **g** RNA FISH assay displayed that circCYP24A1 mainly localized in cytoplasm in ESCC cells

CircRNA expression profiles were analyzed in five paired samples of ESCC based on Illumina sequecing platform. Compared with normal tissues, 5248 circRNAs in ESCC tissue group were significantly up-regulated and 5385 circRNAs were significantly down regulated (Fig. 1a). We focused on those up-regulated circRNAs with relatively high expression, which may be detected in tumor tissues and serum and become targets for treatment and diagnosis. Hsa_circ_0060927 which generated from CYP24A1, designated as circCYP24A1, was the most significantly up-regulated circRNA (with log_2_FoldChange of 5.8451, *P* < 0.0001) in those differentially expressed circRNAs.

CircCYP24A1 is derived from the exons 3–11 of CYP24A1 gene with a length of 1106nt. To verify the circCYP24A1 is a ture circRNA, the backsplice junction site of circCYP24A1 was amplified by using divergent primers and confirmed by Sanger sequencing (Fig. 1b). circCYP24A1 could be amplified by divergent primers, but not convergent primers. There was no predicted product amplified from genomic DNA (Fig. 1b). The expression of circCYP24A1 was verified in two clinical ESCC tissue specimens (Fig. S1). There are different endogenous expression levels of circCYP24A1 in commercial esophageal cancer cell lines (Fig. 1c). PCR analysis showed that circCYP24A1 was more stable than CYP24A1 mRNA after Actinomycin D treatment (Fig. 1d). Additionally, RNase R digestion assay showed that circCYP24A1 was resistant to RNase R, while linear CYP24A1 was degraded after RNase R treatment (Fig. 1f). Then, subcellular localization of circRNA was analyzed. Fluorescence in situ hybridization (FISH) revealed that most of circCYP24A1 localized in cytoplasm (Fig. 1g). Collectively, these results indicate that circCYP24A1 is a bona fide circRNA.

### circCYP24A1 exhibits higher expression in ESCC tissue and its high level indicates poor prognosis

**Fig. 2:**
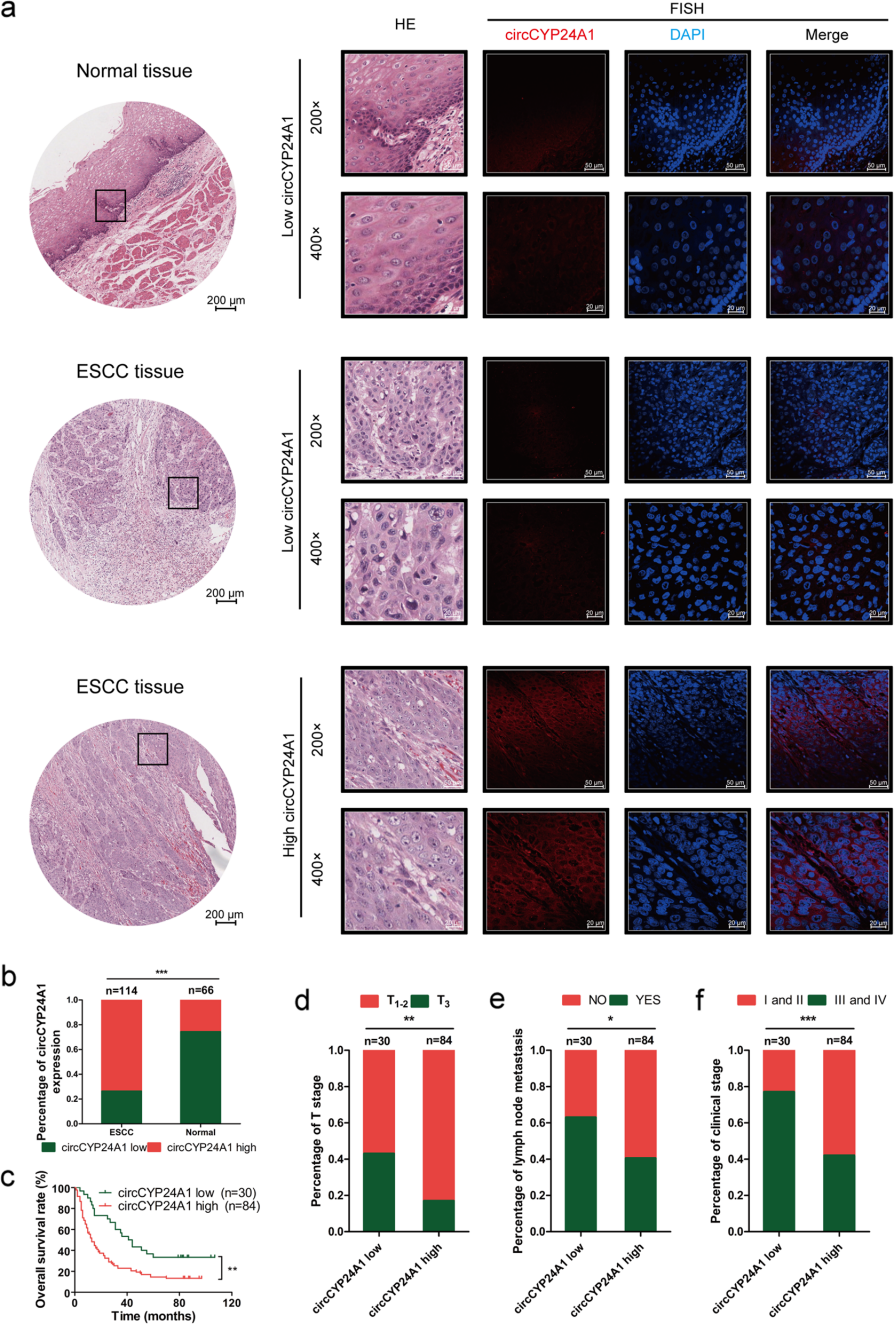
circCYP24A1 exhibits higher expression in ESCC tissue and its high level indicates poor prognosis. **a** The representation pictures of circCYP24A1 expression in 114 ESCC tissues and 66 corresponding adjacent tissues by RNA FISH analysis based on tissue microarray. **b** The percentage of circCYP24A1 expression in 114 ESCC tissues and 66 corresponding adjacent tissues. **c** The correlation between low circCYP24A1 expression and poor patient survival detected by Kaplan–Meier survival analysis. **d** The percentage of T stage in 30 case of ESCC tissues with circCYP24A1 low expression and 84 case of ESCC tissues with circCYP24A1 high expression. **e** The percentage of lymph node metastasis in 30 case of ESCC tissues with circCYP24A1 low expression and 84 case of ESCC tissues with circCYP24A1 high expression. **f** The percentage of clinical stage in 30 case of ESCC tissues with circCYP24A1 low expression and 84 case of ESCC tissues with circCYP24A1 high expression. *P < 0.05, **P < 0.01, ***P < 0.001

In order to detect the expression level of circCYP24A1 in a large population, the tissue microarray including 114 ESCC tissues and 66 corresponding adjacent tissues was selected. It is revealed that circCYP24A1 expression was significantly upregulated in ESCC tissues compared with those corresponding adjacent tissues (Fig. 2a and b, Table S3). The relation between the expression level of circCYP24A1 and clinicopathologic features of ESCC patients was analyzed. It is found that patients with T3, lymph node metastasis and advanced clinical stage exhibited higher expression of circCYP24A1 (Fig. 2d-f, Table S3). Furthermore, the relationship between circCYP24A1 expression and prognosis in ESCC patients was analyzed by Kaplan–Meier analysis and Cox regression model (Fig. 2c, Table S4). Patients with circCYP24A1 high expression had a shorter overall survival. It is revealed that circCYP24A1 high expression and lymph node metastasis were independent prognostic factors of ESCC.

The above results reveal that circCYP24A1 is high expressed in ESCC and its high level indicates poor prognosis, thus promoting us to further explore the biological function in ESCC.

### circCYP24A1 promotes ESCC progression

**Fig. 3:**
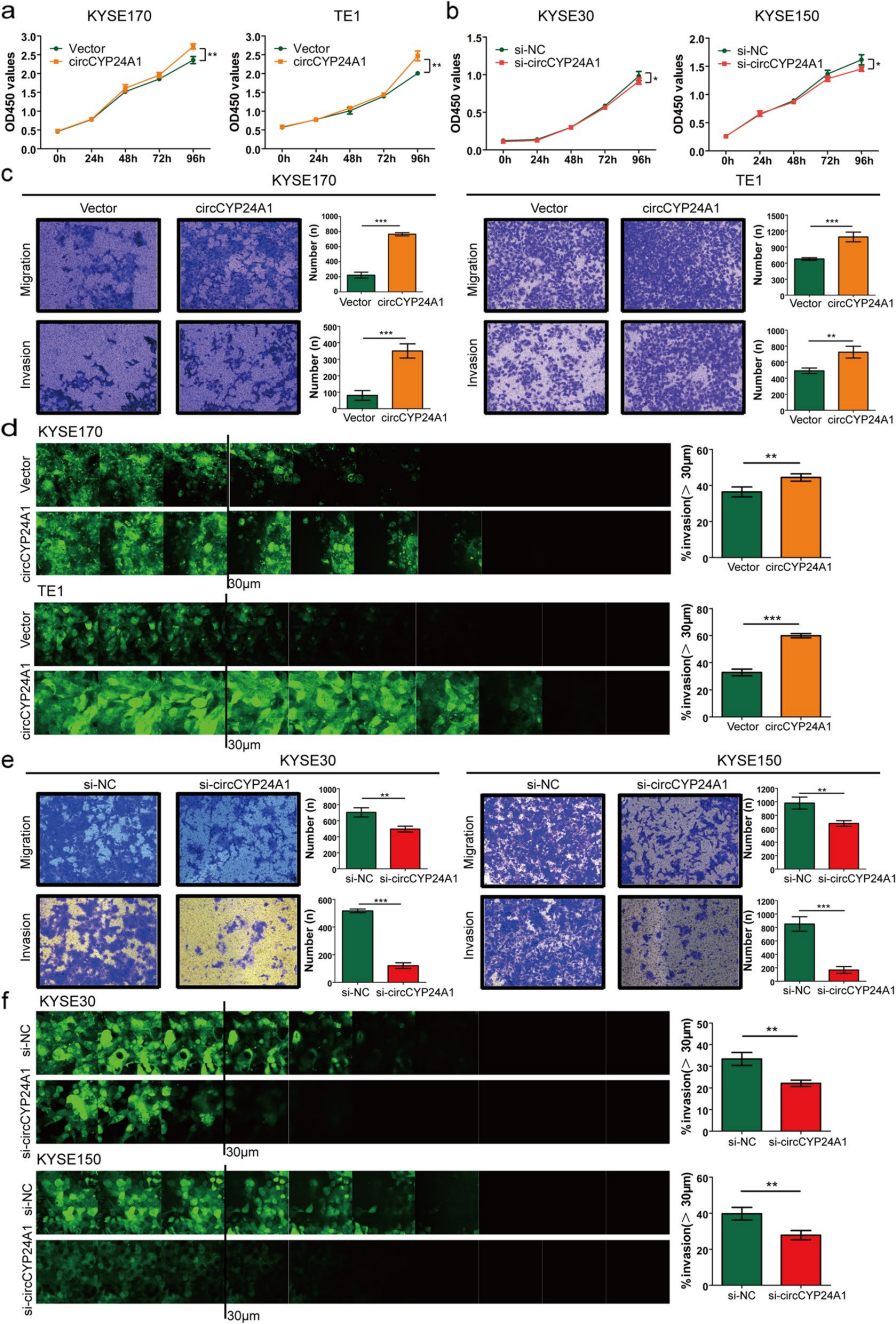
circCYP24A1 accelerates proliferation, migration and invasion of ESCC cells. **a** The proliferation ability of KYSE170 and TE1 cells transfected with circCYP24A1 was detected by CCK-8 assays. **b** The proliferation ability of KYSE30 and KYSE150 cells transfected with si-circCYP24A1 was detected by CCK-8 assays. **c** and **d**, The migration and invasion abilities of KYSE170 and TE1 cells transfected with circCYP24A1 were detected by transwell migration and matrigel invasion assay (**c**) and inverted invasion assay (**d**). E and F, The migration and invasion abilities of KYSE30 and KYSE150 cells transfected with si-circCYP24A1 were detected by transwell migration and matrigel invasion assay (**e**) and inverted invasion assay (**f**). *P < 0.05, **P < 0.01, ***P < 0.001

Gain- and loss-of-function approaches were adopt to explore the biological function of circCYP24A1 in ESCC. We constructed circCYP24A1 over-expression plasmid and verified the transfection efficiency in KYSE170 and TE1 cells as the expression level of circCYP24A1 is relatively low above four cell lines (Fig. S2a). CircCYP24A1 overexpression did not affect the mRNA expression of parental gene (Fig. S2a). These results suggest that CYP24A1 is unaffected by circCYP24A1. CCK8 assay (Fig. 3a) and colony formation assay (Fig. S2c) showed that the proliferation ability of ESCC cells was significantly improved by circCYP24A1 overexpression at 96 h. Further transwell (Fig. 3c), inverted invasion (Fig. 3d) and wound healing assays (Fig. S2e) revealed that circCYP24A1 overexpression promoted the migration and invasion of ESCC cells.

Next, siRNA specifically targeting the backsplice junction region of circCYP24A1 was designed. We transfected siRNA-circCYP24A1 (si-circCYP24A1) into KYSE30 and KYSE150 cells in which the level of circCYP24A1 expression is relatively high in those cell lines. qRT-PCR showed that the level of circCYP24A1 expression decreased significantly by si-circCYP24A1, while the level of CYP24A1 mRNA expression was not change (Fig. S2b). CCK8 assay (Fig. 3b) and colony formation assay (Fig. S2d) revealed that knock down of circCYP24A1 effectively impaired the proliferation abilities of ESCC cells.The inhibitory effects of cell migration and invasion were proved by transwell (Fig. 3e), inverted invasion (Fig. 3f) and wound healing (Fig. S2f) assays.

The above data collectively suggest that circCYP24A1 may promote the pathogenesis and progression of ESCC.

### CCL5 is functional downstream mediator for circCYP24A1

**Fig. 4:**
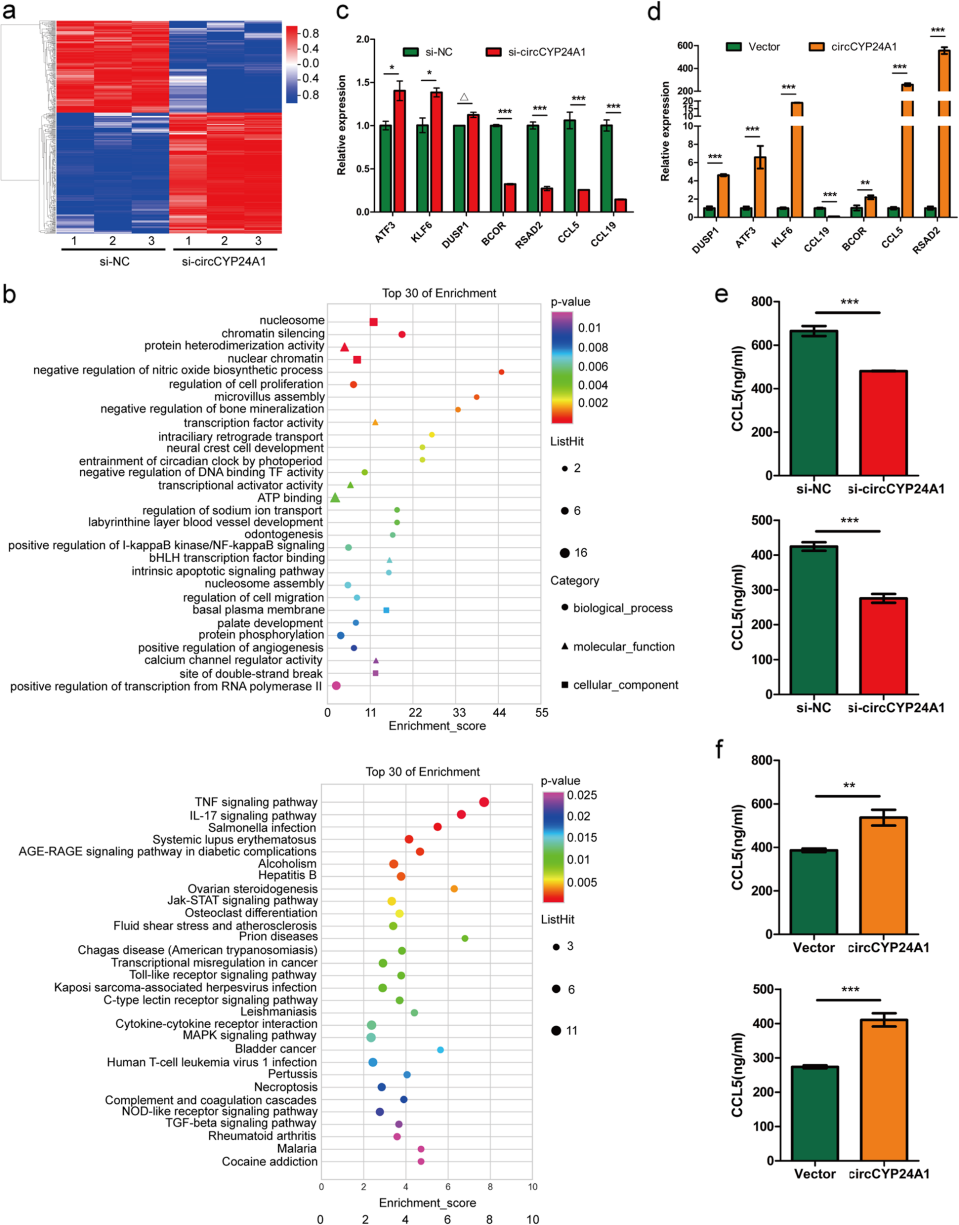
CCL5 is functional downstream mediator for circCYP24A1. **a** 484 changed genes in KYSE150 cells treated with si-circCYP24A1 were identified by Agilent microarray. The screening criteria were the fold change value of up-regulation or down-regulation < 2 and the *P* value < 0.05. **b** Enrichment analysis for representative GO_BP and KEGG pathways in circCYP24A1 target genes. **c** Relative expression levels of representative genes in KYSE150 cells treated with si-circCYP24A1. **d** Relative expression levels of representative genes in TE1 cells transfected with circCYP24A1. **e** ELISA measured the levels of CCL5 in the supernatants of KYSE30 cells upper) and KYSE150 (lower) with circCYP24A1 overexpression. **f** ELISA measured the levels of CCL5 in the supernatants of KYSE170 cells (upper) and TE1 (lower) with circCYP24A1 knockdown. △*P* > 0.05, *P < 0.05,**P < 0.01, ***P < 0.001

To investigate the downstream signaling pathways involved in circCYP24A1, we performed mRNA expression profile microarray for si-NC and si-circCYP24A1 ESCC cells. The results showed that compared with si-NC, 484 genes were changed after si-circCYP24A1 treatment, of which 265 genes were up-regulated and 219 genes were down-regulated (Fig. 4a). Gene ontology biological processes (GO_BP) and KEGG pathways enrichment analysis indicated that circCYP24A1 was involved in regulation of cell proliferation and TNF signaling pathway (Fig. 4b). Some representative genes were selected and validated by qRT-PCR, and CCL5 was one of the most significantly changed genes (Fig. 4c, d). Several studies have found that CCL5 as the chemostatic factors of tumor cells is closely associated with the proliferation, invasion and metastasis [20, 21]. Also, we confirmed that exogenous addition of recombinant human CCL5 (rhCCL5) promoted ESCC cell proliferation, migration and invasion (Fig. S3). Gain- and loss-of-function experments revealed that circCYP24A1 knockdown reduced the level of CCL5 (Fig. 4e), while circCYP24A1 overexpression promoted the level of CCL5, as shown by ELISAs in ESCC cell culture supernatants (Fig. 4f).

**Fig. 5:**
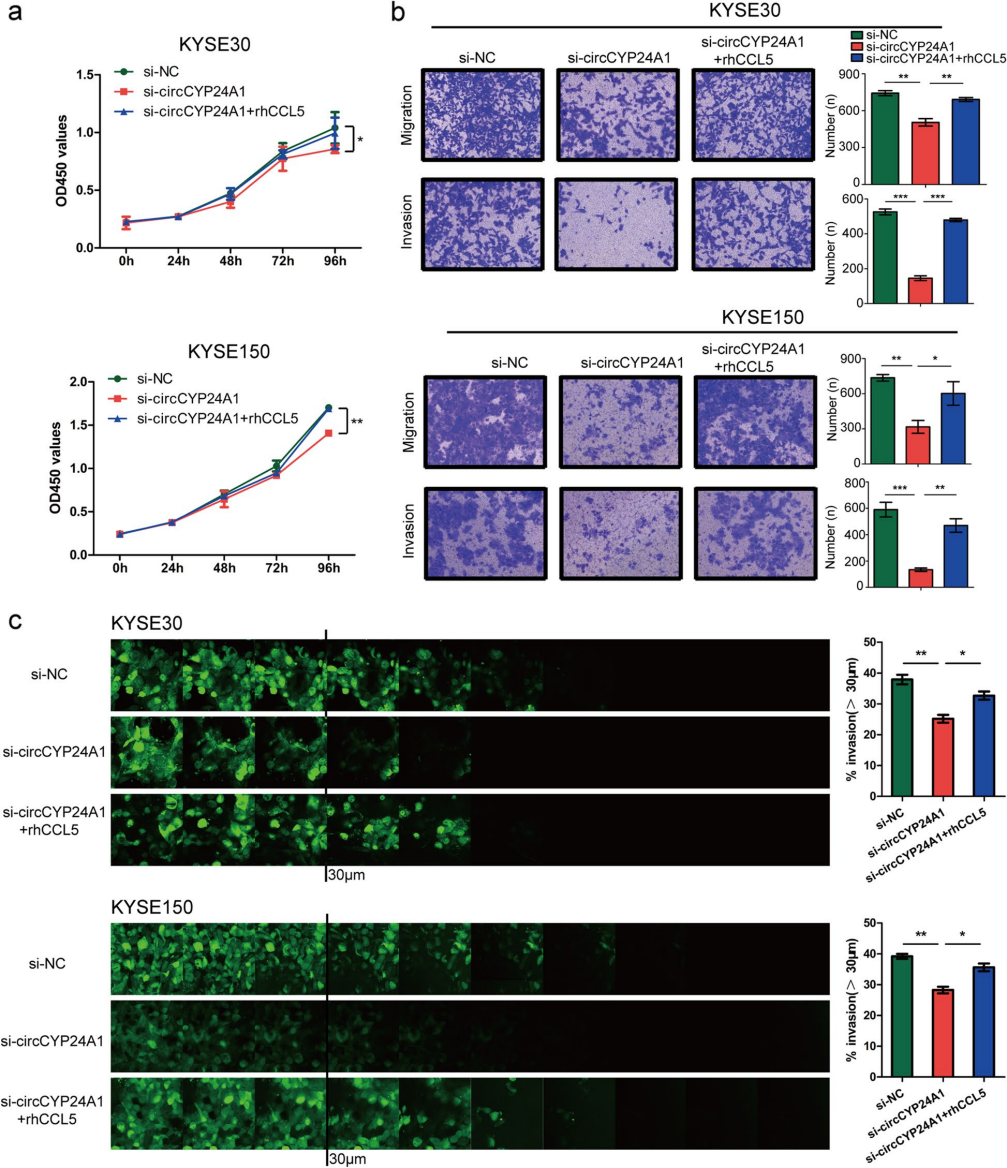
circCYP24A1 enhanced proliferation, migration and invasion of ESCC cells via up-regulating the expression of CCL5. **a** The addition of rhCCL5 partly reversed the inhibition of cell proliferation induced by circCYP24A1 knockdown in KYSE30 and KYSE150 cells. **b** and **c** The addition of rhCCL5 partly reversed the effects of circCYP24A1 knockdown on the ability of migration and invasion. *P < 0.05, **P < 0.01, ***P < 0.001

To confirm whether the biological function of circCYP24A1 on ESCC cells was mediated by stimulating CCL5, the rescue experiments involving circCYP24A1 and CCL5 were carried out. The results revealed that the addition of rhCCL5 partly reversed the inhibition of cell proliferation (Fig. 5a) and colony-forming ability (Fig. S4a, S4b) induced by circCYP24A1 knockdown in ESCC cells. In addition, addition of rhCCL5 partly reversed the effects of circCYP24A1 knockdown on the ability of migration and invasion (Fig. 5b-c, Fig. S4c, S4d). Collectively, these data indicate that circCYP24A1 knockdown inhibits the malignant progression of ESCC partly by reducing CCL5 secreting.

### By interacting with PKM2, circCYP24A1 accelerates CCL5 secretion in vitro and in vivo

**Fig. 6:**
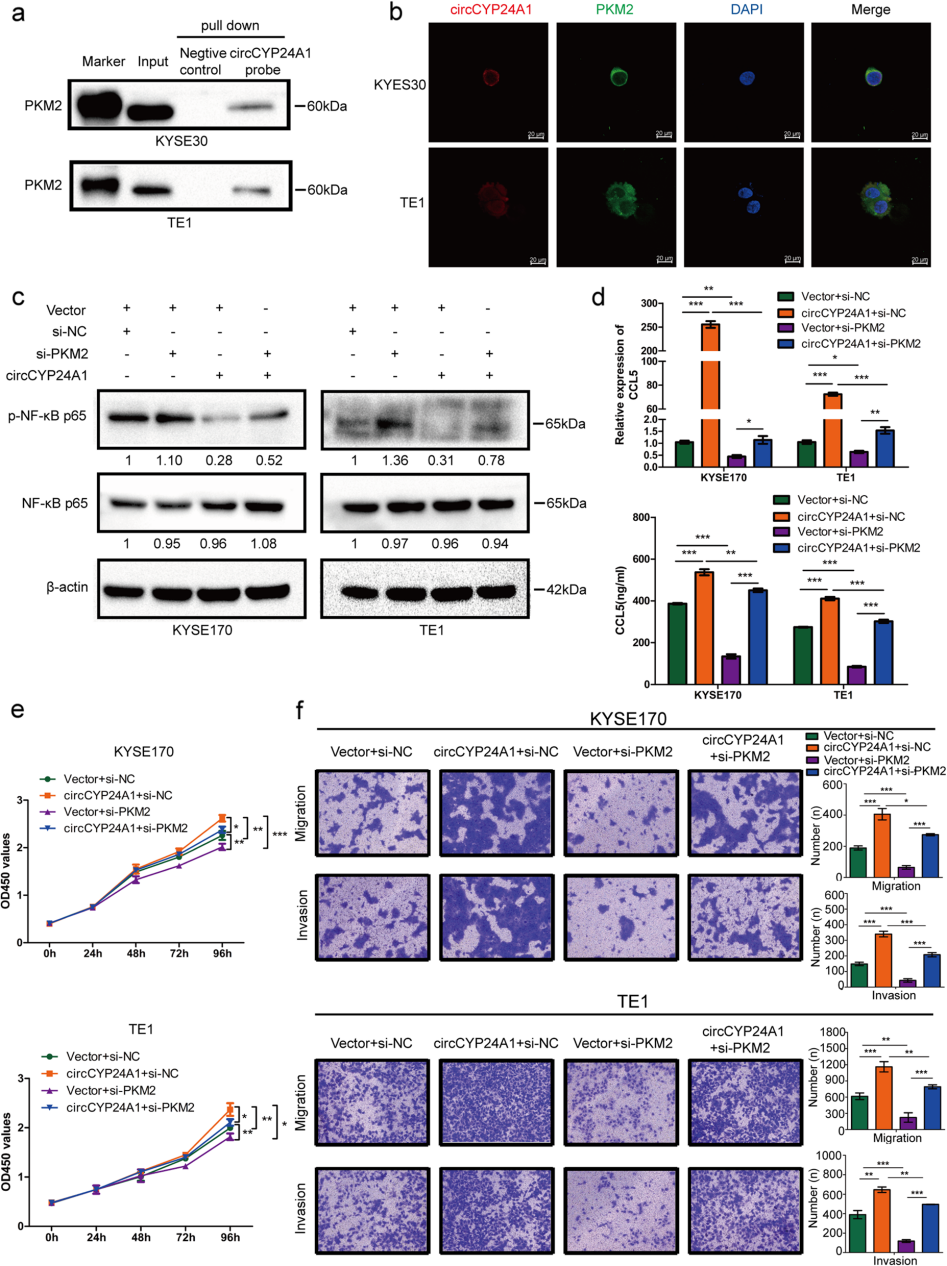
circCYP24A1 interacts with PKM2 and promotes NF-κB pathway-mediated CCL5 secretion in vitro. **a** Binding of CYP24A1 with PKM2 was idntified by RNA pull-down-western blot analysis. b The co-localization of circCYP24A1 (red) with PKM2 (green) in KYSE30 and TE1 cells was idntified by RNA FISH-immunofluorescence. **c** Protein levels in ESCC cells with circCYP24A1 overexpression or PKM2 knockdown. **d** mRNA and protein levels of CCL5 in ESCC cells with circCYP24A1 overexpression or PKM2 knockdown. **e** The proliferation ability of ESCC cells with circCYP24A1 overexpression or PKM2 knockdown was detected by CCK-8 assays. **f** The migration and invasion abilities of ESCC cells with circCYP24A1 overexpression or PKM2 knockdown were detected by transwell migration and matrigel invasion assay. *P < 0.05, **P < 0.01, ***P < 0.001

Then we attempted to explore how circCYP24A1 modulates CCL5 secretion. Bioinformatics Analysis of potential miRNA targets of circCYP24A1 showed that most of them have only one or two binding sites. Therefore, we speculate that circCYP24A1 may play a role through a new mechanism except for miRNA sponge. Recent evidence has suggested that circRNAs are involved in molecular regulation through interaction with protein [22]. To explore the proteins that interact with circCYP24A1, we performed RNA pull-down assay to identify the proteins associated with it. The proteins in RNA pull-down assay were isolated by SDS-PAGE. After coomassie brilliant blue staining, the bands at about 15-130 kDa were excised and analyzed by mass spectrometry. We found that the relatively abundant protein was PKM2 (Table S5), and confirmed this result by using western blot (Fig. 6a). In addition, RNA FISH-immunofluorescence analysis showed that circCYP24A1 and PKM2 had co-localization in the cytoplasm of ESCC cells (Fig. 6b). Previous studies demonstrate that PKM2 could interact with p65/NF-κB and affect the expression of downstream target genes [23, 24]. Our experiments also showed that circCYP24A1 significantly increased the p-p65/NF-κB expression on ESCC cells, and furthermore, the changes were reversed by abrogation of PKM2 (Fig. 6c). Early studies have shown that CCL5 is the downstream target gene of NF-κB [25]. We next hypothesized that such an interaction might contribute to CCL5 secretion, and therefore depletion of PKM2 would affect CCL5 mRNA and protein expression (Fig. 6d) in ESCC cells.

**Fig. 7:**
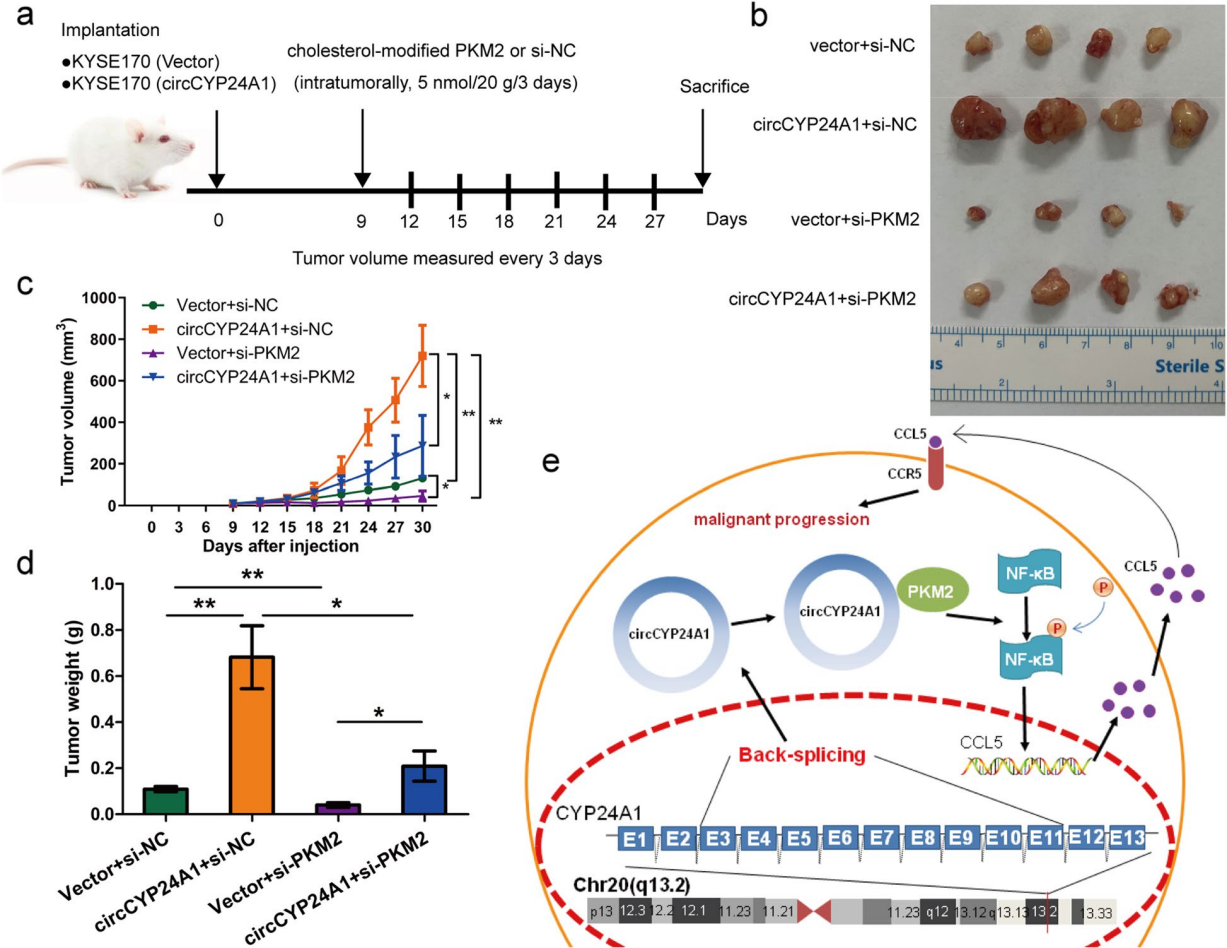
circCYP24A1 interacts with PKM2 and promotes NF-κB pathway-mediated CCL5 secretion in vivo. **a** Schematic diagram of grouping for xenotransplantation model. **b** Representative image of nude mice xenografts in the indicated groups. **c** and **d**, The volume (**c**) and weight (**d**) of subcutaneous xenograft tumors (*n* = 4 mice per group). **e** Proposed model for promotional effects of circCYP24A1C on ESCC progression. *P < 0.05,**P < 0.01

PKM2 knockdown partially recovered the abilities of proliferation (Fig. 6e) and colony formation (Fig. S5a) increased by circCYP24A1 overexpression. Moreover, the inhibitory effects of migration and invasion induced by circCYP24A1 overexpression could be partially restored by PKM2 knockdown (Fig. 6f, Fig. S5a-S5c). Consistent with in vitro experiment, intratumoral injection of si-PKM2 modified by cholesterol significantly restrained the growth of subcutaneous xenografts accelerated by circCYP24A1, including tumor volume and weight (Fig. 7a-d). Furthermore, the expression levels of p-p65/NF-κB were increased in circCYP24A1-overexpressing xenograft tumors, as well as the downstream target gene CCL5, but the expression of the above proteins were partly reverse by intratumoral injection of si-PKM2 modified by cholesterol (Fig. S6).

These data suggest that PKM2-mediated CCL5 secretion may have considerable influence upon the promoting effects of circCYP24A1 on ESCC progression in vitro and in vivo.

## Discussion

Metastasis and progression are the key determinants of poor prognosis for tumor patients. About 90% of cancer deaths are due to metastasis [26]. In the complex process of metastasis, tumor cells encounter a series of obstacles, which require different cell characteristics to successfully colonize distant tissues and organs [26]. As important driving factors in the process of metastasis, cell phenotypes are regulated by epigenetic and transcriptional mechanisms [27]. With the rapid development of RNA-seq technology and biological information analysis, multiple circRNAs have revealed abnormal expression in various tumors and participate in tumor progression through transcriptional and post-transcriptional regulation of gene expression [11–13]. circRNAs possesse high tissue-specificity, conservatism and stability [28], which expected to become potential biomarkers for early diagnosis and prognosis of carcinoma and therapeutic targets. In this study, we analyzed circRNA expression profiles based on RNA-Seq to identified the key circRNAs in ESCC progression. Ultimately, we identified a potential circRNA, termed circCYP24A1, which was up-regulated in ESCC tissues compared with those corresponding adjacent tissues. Recent studies have indicated that circCYP24A1 is abnormal expression in prostate cancer, renal cancer and cutaneous squamous cell carcinoma [29–31]. However, the clinical significance and functional mechanism of circCYP24A1 involved in the progression of ESCC remains obscure.

In this study, we detected the expression level of circCYP24A1 in ESCC patients and analyzed the relationship between the expression and clinicopathologic features. We found that circCYP24A1 expression was significantly higher in ESCC specimens based on tissue microarray than in corresponding adjacent tissues, which was consistent with the results of RNA-seq. However, studies on types of tumors displayed that circCYP24A1 was low expressed in the renal cancer carcinoma [30], which was due to the tissue specificity and disease specificity of circRNA. Multiple studies suggest that circRNA expression was related to the clinicopathological characteristics of tumor patients [6, 8, 18, 30]. We found elevated circCYP24A1 in ESCC tissues was significantly related to larger tumor size and lymph node metastasis as well as advanced clinical stage in ESCC patients. These clinical features indicate the close relationship with the progression of tumor. Moreover, patients with circCYP24A1 high expression had a shorter overall survival. This result revealed that circCYP24A1 may act as a latent tumor promoting factor. Based on the above clinical significance, we further explored the biological function of circCYP24A1 in ESCC through gain- and loss-of-function approaches. We found that circCYP24A1 promoted the ability of proliferation and migration as well as invasion in ESCC cells. We preliminarily clarified the key role of circCYP24A1 in promoting the progress of ESCC.

It is showed that circRNAs participated in tumor progression by activating multiple signal pathways such as β-catenin signaling [6], AMPK-mTOR pathway [8], TGF-β pathway [18], PI3K/AKT/mTOR signaling pathway [29]. To investigate the downstream signaling pathways involved in circCYP24A1, we performed mRNA expression profile microarray and found that circCYP24A1 was involved in regulation of cell proliferation and TNF signaling pathway. Moreover, the downstream target gene CCL5 of circCYP24A1 was selected. Chemokine CCL5 participates in the proliferation and migration of tumor cells, as well as angiogenesis and lymphangiogenesis, by binding to receptors CCR5, CCR3 and CCR1 [32–34]. CCL5 abnormal expression is found in astrocytoma, breast cancer, hepatocellular carcinoma and mutiple other cancers [33, 35, 36]. In this study, we found that the ectopic expression of circCYP24A1 can affect the change of the downstream target gene CCL5, which supported ESCC development. Thus, we demonstrated that circCYP24A1 participates in the malignant progression of ESCC partly by secretion secretion of CCL5.

CircRNA is not evenly distributed in cells and has unique subcellular localization, which determines its cellular functions [11–13, 37]. To make clear the functional mechanism, the subcellular localization of circCYP24A1 in ESCC cells was detected and FISH revealed that most of circCYP24A1 localized in cytoplasm. CircRNAs located in the cytoplasm could acts as ceRNAs competitively bind miRNAs [18, 29, 30] and bind to RBPs [6, 8, 18, 19]. A few cytoplasmic circRNAs also have the ability of translating novel polypeptides or proteins [16, 38, 39]. In ESCC, most studies concentrate on the function of circRNAs as miRNA sponges [40, 41]. Because of the fewer miRNA binding sites of circCYP24A1, we speculate that circCYP24A1 may play a role through a new mechanism except for miRNA sponge. RBPs play a essential role in gene expression and post-transcriptional gene regulation (PTGR), especially in the occurrence and development of cancer [42]. In order to elucidate the the molecular mechanism of up-regulated circCYP24A1 in ESCC, we analyzed the potential RBPs of circCYP24A1. We found that PKM2 could directly bind to circCYP24A1 and induce its biological function in ESCC. In addition to the glycolytic function, PKM2 plays a vital role in regulating gene expression and cell cycle progression [43]. It is reported that PKM2 acts as a coactivator of HIF-1α to regulating glucose metabolism in cancer cells [44]. Recently, investigations suggested that miRNAs, long non-coding RNAs (lncRNAs), and circRNAs regulate the biological behaviors by targeting PKM2. By directly binding to the 3'UTR region of PKM2, miRNAs can effectively regulate a variety of metabolic and biological processes [45, 46]. LncRNA-FEZF1-AS1 could bind with PKM2 and increase its stability and expression [47]. Up to now, lots of studies suggested that circRNAs can sponge miRNAs and regulate the expression of PKM2. For instance, upregulated circSHKBP1 promoted the progression of non-small cell lung cancer via the miR-1294/PKM2 axis [48]. In hypoxia state, circMAT2B enhanced glycolysis of hepatocellular carcinoma cells by activating circMAT2B/miR-338-3p/PKM2 axis [49]. However, few literatures analysed that PKM2 acts as RBPs in the mechanism research of circRNAs. This study may promote further research on the the biological functions of circRNA through its RBPs. Previous studies demonstrate that PKM2 could interact with p65/NF-κB and affect the expression of downstream target genes in breast cancer and hypoxic pancreatic tumors [23, 24], while CCL5 was proved to be a downstream target gene of the NF-κB pathway [25]. Herein, we confirmed that circCYP24A1 significantly increased the p-p65/NF-κB expression, and the changes were reversed by abrogation of PKM2, as well as CCL5 mRNA and protein. Forthermore, we identified the circCYP24A1/ PKM2/ NF-κB/ CCL5 axis in ESCC by performing a series of rescue experiments. Subsequently, we explored the function of circCYP24A1 in ESCC progression in vivo through the modeling of human ESCC xenograf. As expected, intratumoral injection of si-PKM2 significantly restrained the growth of subcutaneous xenografts accelerated by circCYP24A1. Consequently, we conclude that circCYP24A1 interacts with PKM2 to promote the phosphorylation of NF-κB, which promotes the secretion of CCL5 and accelerate malignant progression of ESCC (Fig. 7e). These data indicated that circCYP24A1 might be a potential effective biomarker for judging prognosis and a therapeutic target in ESCC.

## Conclusion

In summary, this study puts forward a potential mechanism of regulating ESCC malignant progression through the circCYP24A1/ PKM2/ NF-κB/ CCL5 pathway, which may provied a new approach and the therapeutic target for judging prognosis and therapy of ESCC. We deepened the understanding of the role of circRNAs in the progression of ESCC. In the future, we will clarify that whether circCYP24A1/ PKM2/ NF-κB could affect tumor microenvironment by promoting the paracrine and distal secretion of CCL5.

## Supplementary Information

**Additional file 1.** Supplementary figure.

**Additional file 2.** Supplementary table.

## Data Availability

All data and materials in our study are available upon reasonable request.

## Abbreviations

EC: Esophageal carcinoma
ESCC: Esophageal squamous cell carcinoma
ncRNA: Non-coding RNA
CCL5: Chemokine (C-Cmotif) ligand 5
circRNAs: Circular RNAs
ceRNAs: Competitive endogenous RNAs
miRNAs: MicroRNAs
RBPs: RNA binding proteins
CLIP: Cross-linked immunoprecipitation
CYP24A1: Cytochrome P450 family 24 subfamily A member 1
PKM2: M2 isoform of pyruvate kinase
FISH: Fluorescence in situ hybridization
CCK-8: Cell Counting Kit-8
ELISA: Enzyme-linked-immunosorbent serologic assay
ECL: Electrochemiluminescence
rhCCL5: Recombinant human CCL5
IACUC: Institutional Animal Care and Use Committee
GO_BP: Gene ontology biological processes
PTGR: Post-transcriptional gene regulation
lncRNAs: Long non-coding RNAs
