# Correction: circCYP24A1 facilitates esophageal squamous cell carcinoma progression through binding PKM2 to regulate NF-κB-induced CCL5 secretion

**DOI:** 10.1186/s12943-023-01730-0

**Published:** 2023-01-26

**Authors:** Lina Gu, Yang Sang, Xixi Nan, Yang Zheng, Fei Liu, Lingjiao Meng, Meixiang Sang, Baoen Shan

**Affiliations:** 1 Research Center, the Fourth Hospital of Hebei Medical University, 050017 Shijiazhuang, Hebei People’s Republic of China; 2 Animal Center, the Fourth Hospital of Hebei Medical University, Shijiazhuang, Hebei People’s Republic of China; 3 Tumor Research Institute, the Fourth Hospital of Hebei Medical University, 050017 Shijiazhuang, Hebei People’s Republic of China

**Correction: Mol Cancer 21, 217 (2022)**

**https://doi.org/10.1186/s12943-022-01686-7**

Following publication of the original article [1], errors were identified in the images presented in Figs. 2 and 6; specifically:

- Fig. 2d-f The colors of the legend were reversed. The correct representative images are now used.
- Fig. 6c The grouping symbols were mislabeled. The correct representative images are now used.

**Fig. 2:**
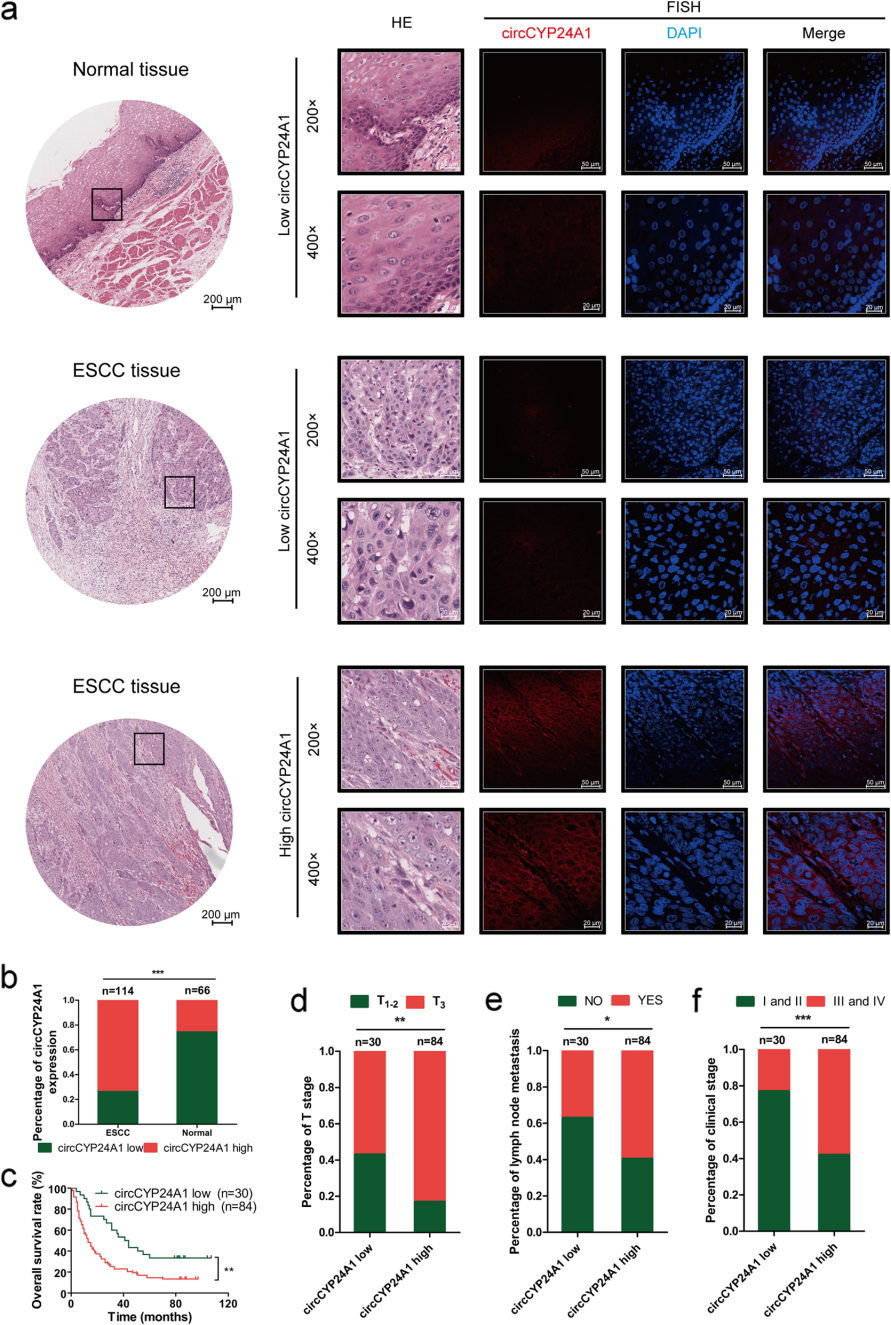
circCYP24A1 exhibits higher expression in ESCC tissue and its high level indicates poor prognosis. **a** The representation pictures of circCYP24A1 expression in 114 ESCC tissues and 66 corresponding adjacent tissues by RNA FISH analysis based on tissue microarray. **b** The percentage of circCYP24A1 expression in 114 ESCC tissues and 66 corresponding adjacent tissues. **c** The correlation between low circCYP24A1 expression and poor patient survival detected by Kaplan–Meier survival analysis. **d** The percentage of T stage in 30 case of ESCC tissues with circCYP24A1 low expression and 84 case of ESCC tissues with circCYP24A1 high expression. **e** The percentage of lymph node metastasis in 30 case of ESCC tissues with circCYP24A1 low expression and 84 case of ESCC tissues with circCYP24A1 high expression. **f** The percentage of clinical stage in 30 case of ESCC tissues with circCYP24A1 low expression and 84 case of ESCC tissues with circCYP24A1 high expression. *P < 0.05, **P < 0.01, ***P < 0.001

**Fig. 6:**
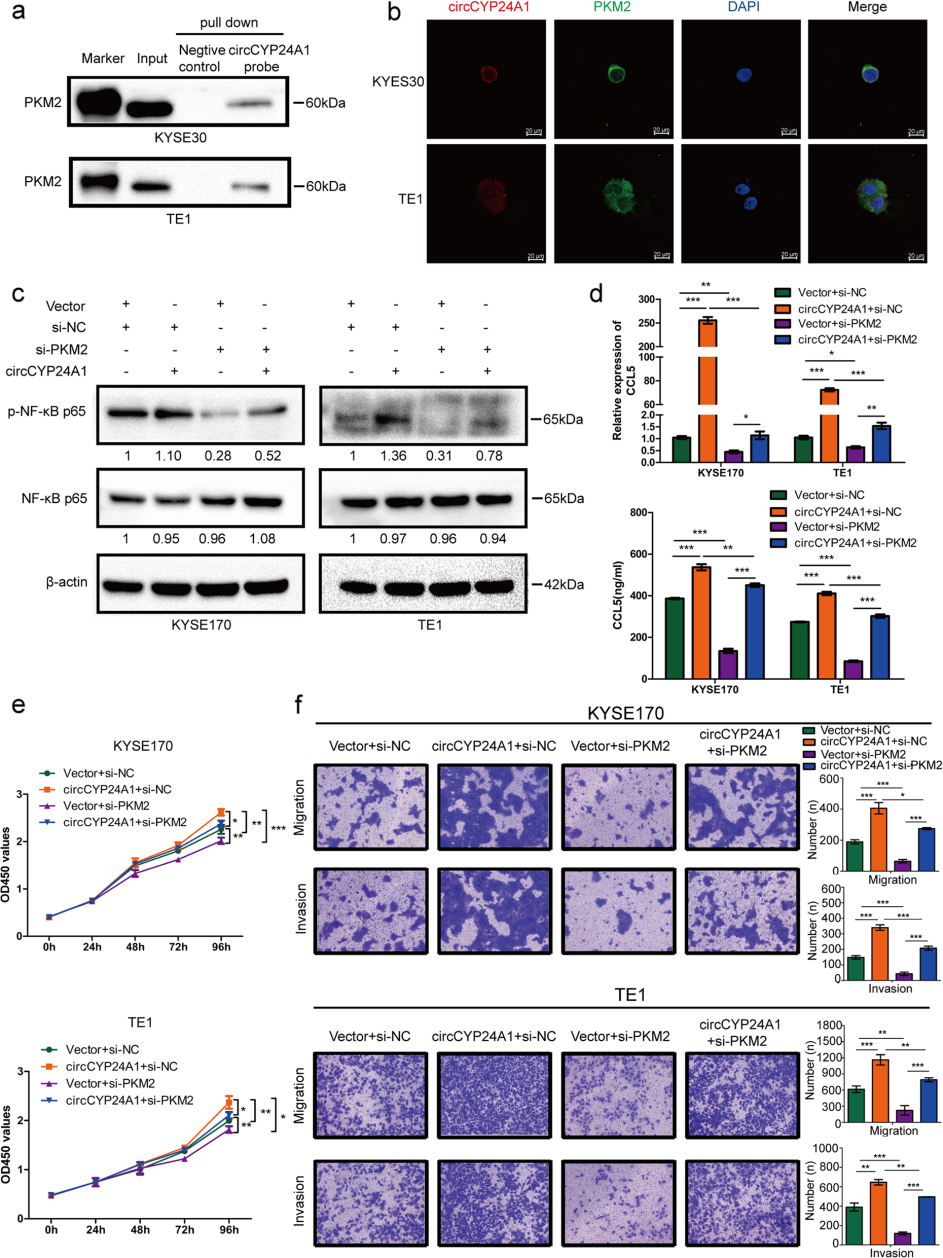
circCYP24A1 interacts with PKM2 and promotes NF-κB pathway-mediated CCL5 secretion in vitro. **a** Binding of CYP24A1 with PKM2 was idntified by RNA pull-down-western blot analysis. **b** The co-localization of circCYP24A1 (red) with PKM2 (green) in KYSE30 and TE1 cells was idntified by RNA FISH-immunofluorescence. **c** Protein levels in ESCC cells with circCYP24A1 overexpression or PKM2 knockdown. **d** mRNA and protein levels of CCL5 in ESCC cells with circCYP24A1 overexpression or PKM2 knockdown. **e** The proliferation ability of ESCC cells with circCYP24A1 overexpression or PKM2 knockdown was detected by CCK-8 assays. **f** The migration and invasion abilities of ESCC cells with circCYP24A1 overexpression or PKM2 knockdown were detected by transwell migration and matrigel invasion assay. *P < 0.05, **P < 0.01, ***P < 0.001

The corrected figures are provided here. The correction does not have any effect on the results or conclusions of the article.
