# Supplementary material for: circCYP24A1 facilitates esophageal squamous cell carcinoma progression through binding PKM2 to regulate NF-κB-induced CCL5 secretion
Source: Mol Cancer. 2022 Dec 13;21:217. doi: 10.1186/s12943-022-01686-7 (PMC9746112; doi:10.1186/s12943-022-01686-7)
Supplement: Supplementary file 1 — Additional file 1. Supplementary figure. [file 12943_2022_1686_MOESM1_ESM.docx]

**Supplementary Figure**

**
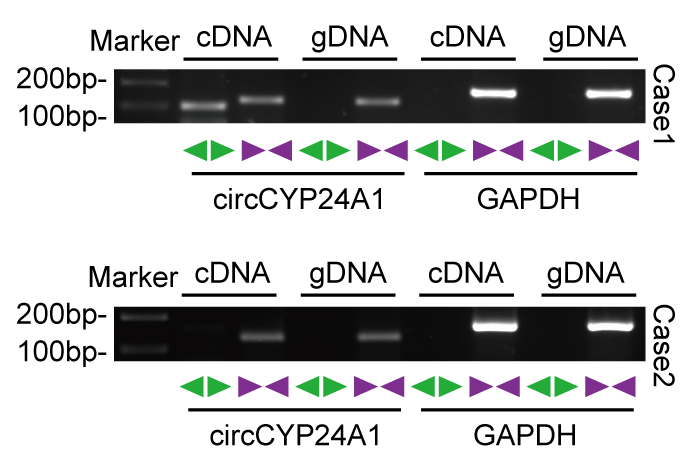
**

**Supplementary Figure 1. The expression of circCYP24A1 in two clinical ESCC tissue specimens was detected by RT-PCR.**

**
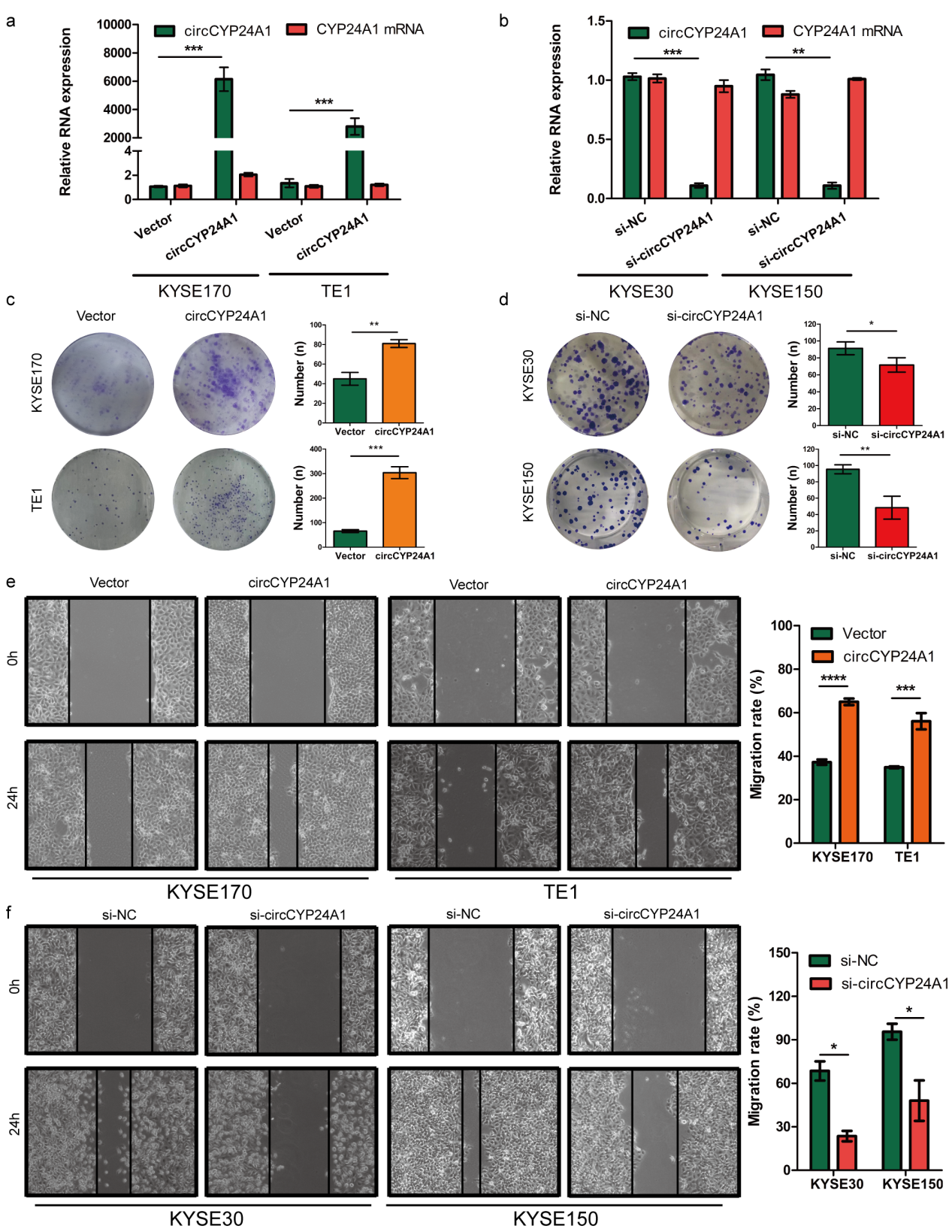
**

**Supplementary Figure 2. The effect of circCYP24A1 on clonogenic ability and cell migration.** a, The expression of circCYP24A1 and linear CYP24A1 in circCYP24A1 low-expressed KYSE170 and TE1 cells transfected with overexpression vector circCYP24A1. b, The expression of circCYP24A1 and linear CYP24A1 in circCYP24A1 high-expressed KYSE30 and KYSE150 cells transfected with si-circCYP24A1. c, The clonogenic ability of KYSE170 and TE1 cells transfected with circCYP24A1 was detected by colony formation assays. d, The clonogenic ability of KYSE30 and KYSE150 cells transfected with si-circCYP24A1 was detected by colony formation assays. e, The cell migration of KYSE170 and TE1 cells transfected with circCYP24A1 was detected by wound healing assays. f, The cell migration of KYSE30 and KYSE150 cells transfected with si-circCYP24A1 was detected by wound healing assays. *P < 0.05,**P < 0.01, ***P < 0.001.

**
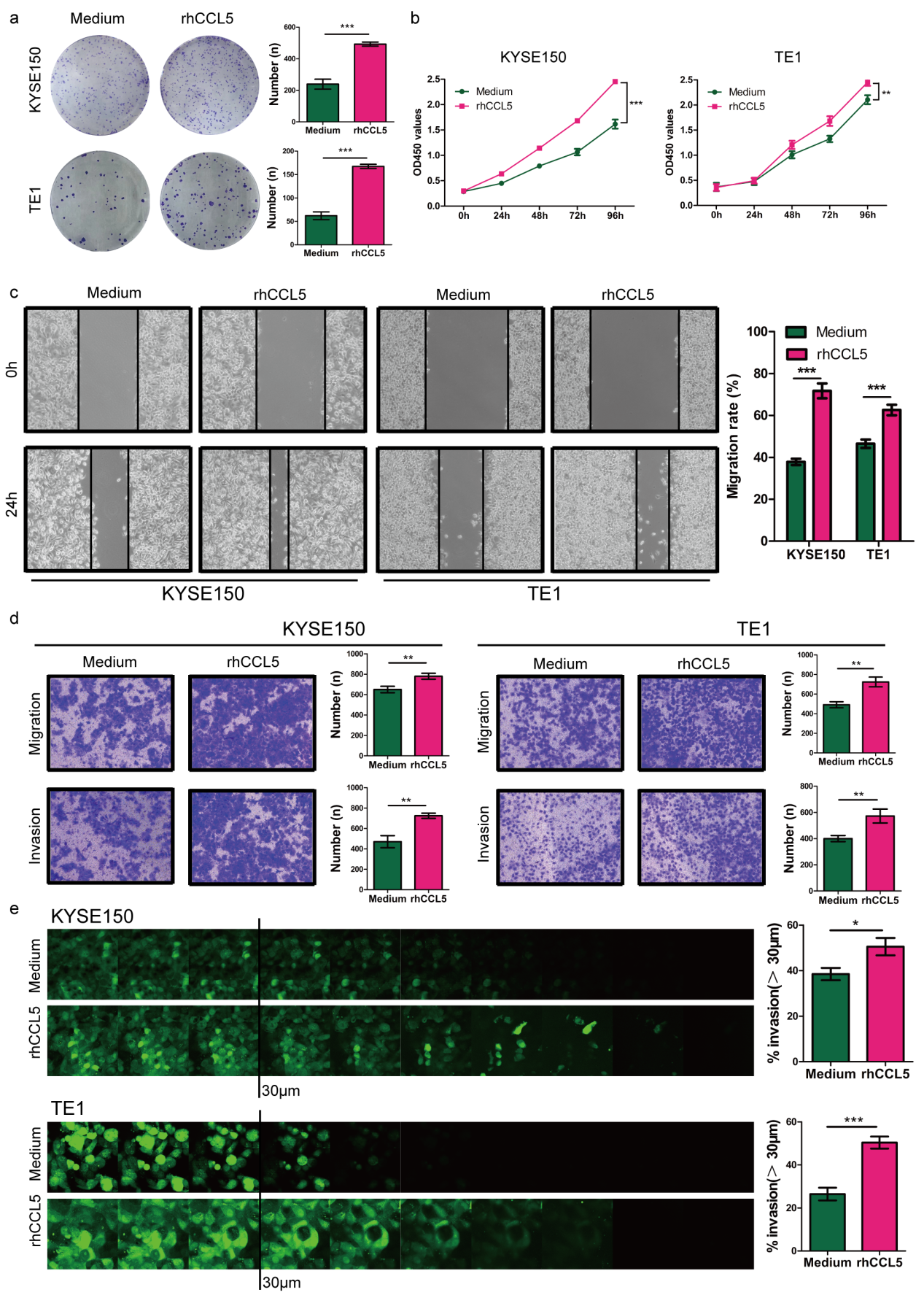
**

**Supplementary Figure 3.** **The effect of rhCCL5 on** **abilities of proliferation, migration and invasion in ESCC cells.** a and b, The proliferation ability of KYSE150 and TE1 cells with or without rhCCL5 was detected by colony formation assays (a) and CCK-8 assays (b). c, d and e, The cell migration and invasion of KYSE150 and TE1 cells with or without rhCCL5 was detected by wound healing assays (c), transwell assays (d) and inverted invasion assay (e). **P < 0.01, ***P < 0.001.

**
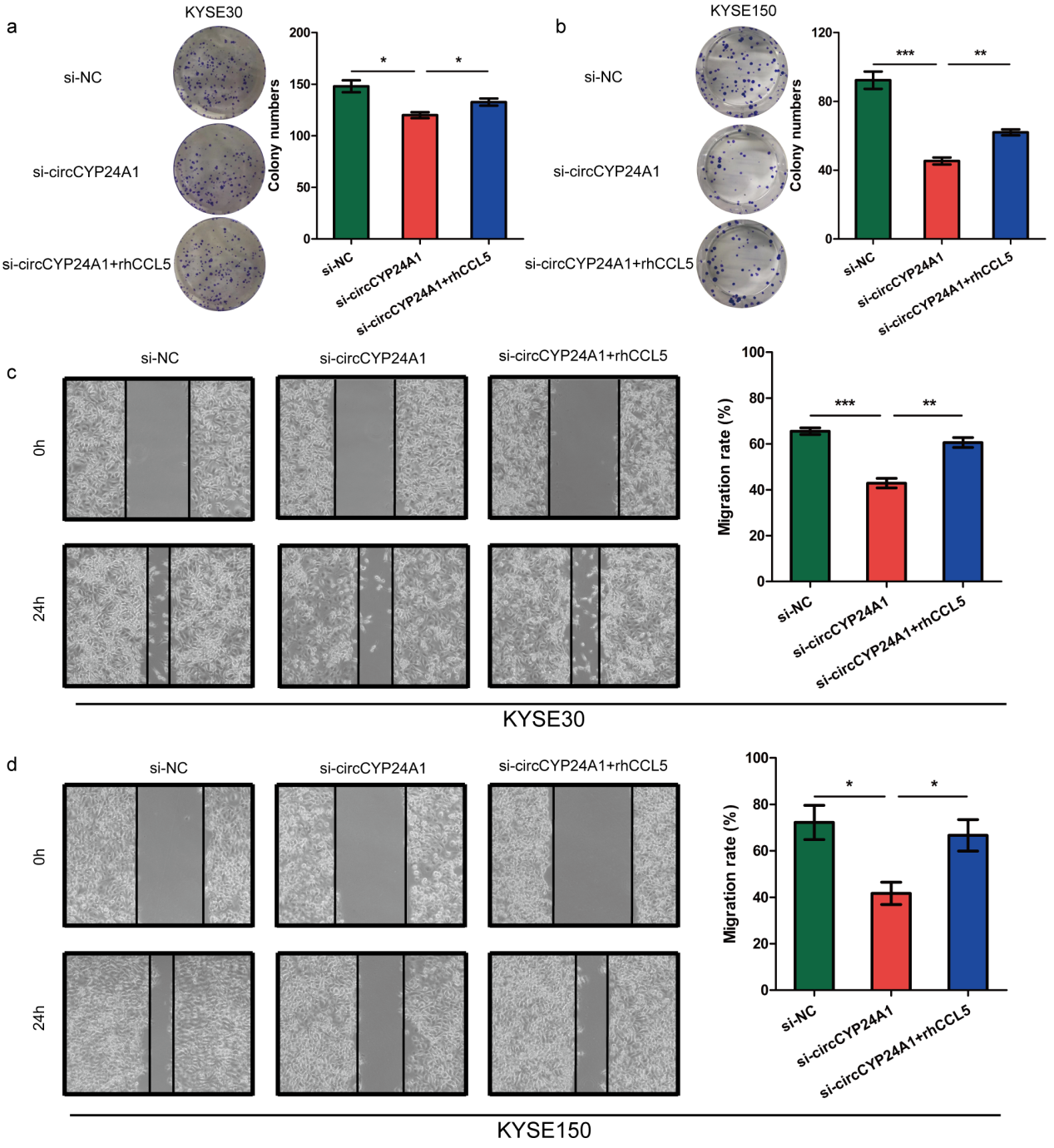
**

**Supplementary Figure 4. The addition of rhCCL5 partly reversed the inhibition of clonogenic ability and cell migration induced by circCYP24A1 knockdown in KYSE30 and KYSE150 cells.** a and b, The addition of rhCCL5 partly reversed the inhibition of clonogenic ability induced by circCYP24A1 knockdown in KYSE30 (a) and KYSE150 (b) cells. c and d, The addition of rhCCL5 partly reversed the inhibition of cell migration induced by circCYP24A1 knockdown in KYSE30 (c) and KYSE150 (d) cells. *P < 0.05,**P < 0.01, ***P < 0.001.

**
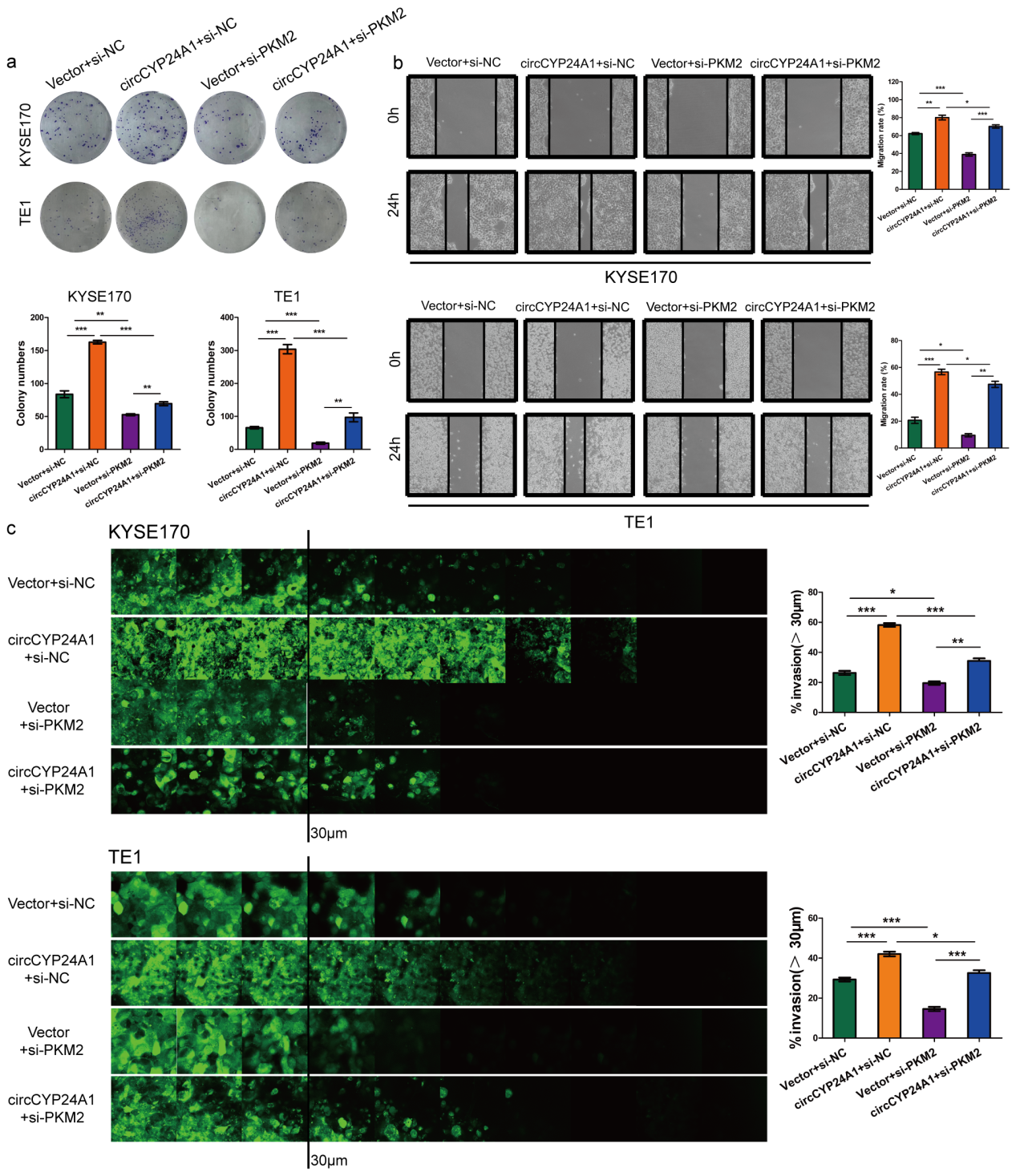
**

**Supplementary Figure 5. PKM2 knockdown rescued circCYP24A1-mediated accelerating effect of clonogenic ability, cell migration and invasion in ESCC cells.** a, PKM2 knockdown partly reversed the inhibition of clonogenic ability induced by circCYP24A1 overexpression in KYSE170 and TE1 cells. b, PKM2 knockdown partly reversed the inhibition of cell horizontal migration induced by circCYP24A1 knockdown in KYSE170 and TE1 cells. c, PKM2 knockdown partly reversed the inhibition of vertical invasion induced by circCYP24A1 knockdown in KYSE170 and TE1 cells. *P < 0.05,**P < 0.01, ***P < 0.001.


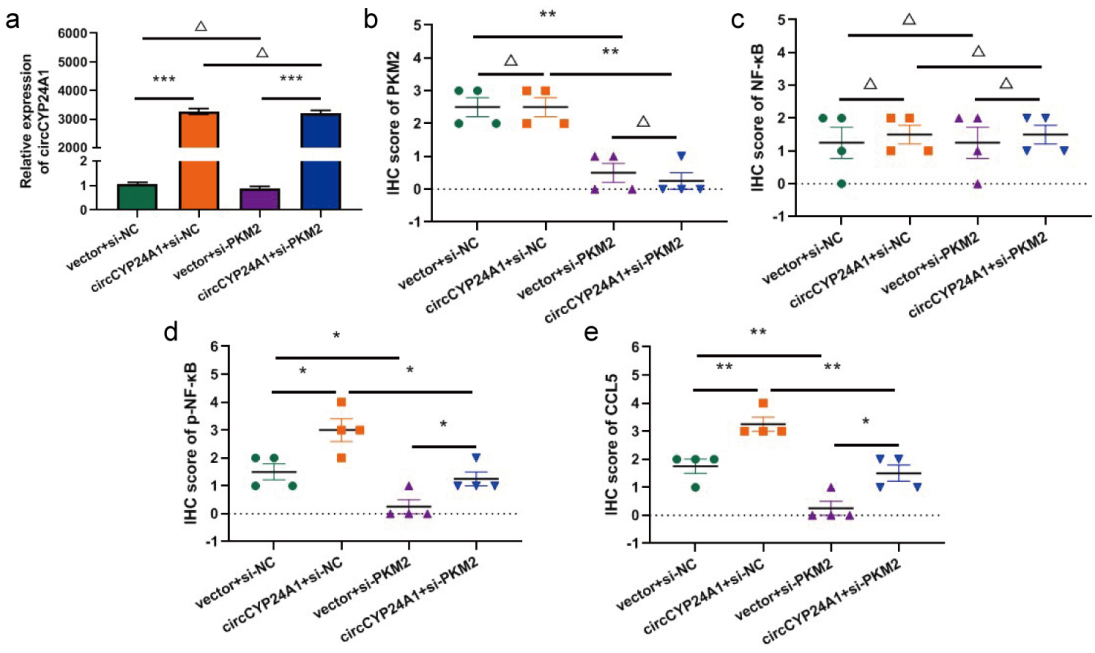


**Supplementary Figure 6. PKM2 knockdown rescued circCYP24A1-mediated NF-κB pathway activation and secretion of CCL5 in vivo.** a, The expression of circCYP24A1 in xenograft tumors detected by qRT-PCR. b-e, IHC scores of PKM2 (b), NF-κB (c), p-NF-κB (d) and CCL5 (e) in xenograft tumors. △P＞0.05, *P < 0.05, **P < 0.01, ***P < 0.001.
