# Supplementary material for: circCYP24A1 facilitates esophageal squamous cell carcinoma progression through binding PKM2 to regulate NF-κB-induced CCL5 secretion
Source: Mol Cancer. 2022 Dec 13;21:217. doi: 10.1186/s12943-022-01686-7 (PMC9746112; doi:10.1186/s12943-022-01686-7)
Supplement: Supplementary file 2 — Additional file 2. Supplementary table. [file 12943_2022_1686_MOESM2_ESM.docx]

**Supplementary Table S1 Patient Information of ESCC patients for RNA-Seq**

| **Sample ID** | **Age** | **gender** | **Histological grade** | **Clinical stage** | **T stage** | **Lymph node metastasis** | **Distant metastasis** | **Histological type** |
| --- | --- | --- | --- | --- | --- | --- | --- | --- |
| 1 | 65 | Male | II | III | T3 | Yes | No | ESCC |
| 2 | 63 | Male | III | IV | T3 | Yes | No | ESCC |
| 3 | 70 | Female | II | III | T3 | Yes | No | ESCC |
| 4 | 53 | Female | II | II | T2 | No | No | ESCC |
| 5 | 64 | Female | II | II | T3 | No | No | ESCC |

**Supplementary Table S2 Primer sequences and reaction conditions used in this study**

| Experiment types | Gene | Primers | Annealing temperature (℃) | Product size(bp) |
| --- | --- | --- | --- | --- |
| RT-PCR and RT-qPCR | circCYP24A1 |  |  |  |
|  | Divergent primer | F:5’- CGTTTTGCCAGCGATAATACG -3’ | 58 | 102 |
|  |  | R:5’- AGCTTCATCACTTCCCCTGG -3’ |  |  |
|  | Convergent primers | F:5’- AATGGGCAGCCGTTAGGAAA -3’ | 58 | 131 |
|  |  | R:5’- TATTTGCGGACAATCCAACA -3’ |  |  |
|  | PKM2 | F:5’- TGACGAGAACATCCTGTGGC -3’ | 58 | 141 |
|  |  | R:5’- TTTTCCACCTCCGTCACCAG -3’ |  |  |
|  | ATF3 | F:5’- AAGGATTTTCAGCACCTTGCC -3’ | 58 | 161 |
|  |  | R:5’- TTCCTTGACAAAGGGCGTCA -3’ |  |  |
|  | KLF6 | F:5’- GAGCCCTGCTATGTTTCAGC -3’ | 58 | 223 |
|  |  | R:5’- AAAGTTCCTCGGAGCTGTCA -3’ |  |  |
|  | DUSP1 | F:5’- TGCCTTGATCAACGTCTCAG -3’ | 58 | 159 |
|  |  | R:5’- ACCCTTCCTCCAGCATTCTT -3’ |  |  |
|  | BCOR | F:5’- AAACGCCGACGAGTCTCTAA -3’ | 58 | 236 |
|  |  | R:5’- TCTTCCGACCAGCTTCTGTT -3’ |  |  |
|  | RSAD2 | F:5’- CTTGAACCCTGAAGGCAGAG -3’ | 58 | 152 |
|  |  | R:5’- GTGTCCTTGGGCTTTCACAT -3’ |  |  |
|  | CCL5 | F:5’- GAGGCTTCCCCTCACTATCC -3’ | 58 | 155 |
|  |  | R:5’- CTCAAGTGATCCACCCACCT -3’ |  |  |
|  | CCL19 | F:5’- TGCCTGCTGTAGTGTTCACC -3’ | 58 | 218 |
|  |  | R:5’- GGTCCTTCCTTCTGGTCCTC -3’ |  |  |
|  | GAPDH | F:5’- TGGCTTCAGGAGAAGGAAAA -3’ | 58 | 168 |
|  |  | R:5’- GCGCCCAATACGACCAAATC -3’ |  |  |
| pC5-seq | circCYP24A1 | F:5’- TGTGAATTTGACCCTTAAGA -3’ |  |  |
|  |  | R:5’- TCCTCTCTTGATTTCCTTATT -3’ |  |  |
| FISH | circCYP24A1 | 5’-biotin-TCTTCCCCTTCCCTGAGGCGTATTA-3’-biotin |  |  |
| RNA pull-down | circCYP24A1 | 5’-CTTCCCTGAGGCGTATTATC-/3bio/-3’ |  |  |

F: Forward primer; R: Reverse primer.

**Supplementary Table S3 Relationship between the expression level of**

**circCYP24A1 and clinical pathological features in ESCC patients**

| Group | N | circCYP24A1 | | | *χ^2^* | *P* |
| --- | --- | --- | --- | --- | --- | --- |
|  |  | low | | high |  |  |
| Age | | | | | 0.022 | 0.882 |
| ≤60 | 33 | 9 | 24 | |  |  |
| >60 | 81 | 21 | 60 | |  |  |
| Gender | | | | | 2.250 | 0.134 |
| Male | 84 | 19 | 65 | |  |  |
| Female | 30 | 11 | 19 | |  |  |
| Pathological differentiation | | | | | 0.025 | 0.874 |
| I and II | 47 | 12 | 35 | |  |  |
| III | 67 | 18 | 49 | |  |  |
| T stage | | | | | 15.599 | ＜0.001 |
| T_1-2_ | 27 | 15 | | 12 |  |  |
| T_3_ | 87 | 15 | | 72 |  |  |
| Lymph node metastasis | | | | | 4.642 | 0.031 |
| No | 53 | 19 | 34 | |  |  |
| Yes | 61 | 11 | 50 | |  |  |
| Clinical stage | | | | | 17.161 | ＜0.001 |
| I and II | 58 | 25 | 33 | |  |  |
| III | 56 | 5 | 51 | |  |  |

**Supplementary Table S4 Univariate and multivariable analyses of prognostic factors in ESCC**

| Variable | Univariate analysis | | |  | Multivariate analysis | | |
| --- | --- | --- | --- | --- | --- | --- | --- |
|  | HR | *P* value | 95% CI |  | HR | *P* value | 95% CI |
| Expression of circCYP24A1  High vs Low | 4.173 | ＜0.001 | 2.332-7.470 |  | 3.702 | ＜0.001 | 2.145-7.517 |
| Gender  Male vs Female | 2.177 | 0.003 | 1.295-3.658 |  | 1.689 | 0.052 | 0.995-2.868 |
| Age(years)  ＜60 vs ≥60 | 1.062 | 0.792 | 0.678-1.664 |  |  |  |  |
| Histological grade  I and II vs III | 1.006 | 0.976 | 0.664-1.525 |  |  |  |  |
| T stage  T_1-2_ vs T_3_ | 2.555 | 0.001 | 1.497-4.361 |  | 1.682 | 0.073 | 0.953-2.968 |
| Lymph node metastasis  No vs Yes | 1.800 | 0.006 | 1.184-2.737 |  | 1.694 | 0.021 | 1.083-2.649 |
| Clinical stage  I and II vs III | 3.038 | ＜0.001 | 1.952-4.730 |  |  |  |  |

**Supplementary Table S5 List of the 30 most abundant proteins coprecipitated with circCYP24A1 identified by MS.**

| Gene name | Abundances | MW [kDa] |
| --- | --- | --- |
| HNRNPM | 250812213.15625 | 77.5 |
| ALB | 178644135.203125 | 69.3 |
| HNRNPD | 155402490.625 | 38.4 |
| HNRNPK | 122031499.890625 | 50.9 |
| PC | 71453370.1875 | 129.6 |
| TF | 34206882.0273438 | 77 |
| HNRNPA2B1 | 30199283.28125 | 37.4 |
| HNRNPA1 | 29464801.21875 | 38.7 |
| PCBP1 | 24039560.1875 | 37.5 |
| SFPQ | 21498406.90625 | 76.1 |
| PCBP2 | 19565862 | 38.6 |
| HSPA5 | 16710444.078125 | 72.3 |
| ATP5F1A | 15545304.96875 | 59.7 |
| DSG1 | 14068136.796875 | 113.7 |
| HSPA9 | 11772313.71875 | 73.6 |
| JUP | 11647001.7578125 | 81.7 |
| HSPD1 | 10538214.453125 | 61 |
| RPA2 | 9375088.28125 | 29.2 |
| DSC1 | 8945592.5 | 99.9 |
| SERPINA1 | 8387983.921875 | 46.7 |
| PKM2 | 8079740.84375 | 57.9 |
| TIAL1 | 7709833.90625 | 41.6 |
| RBM4B | 7685728.6875 | 40.1 |
| YWHAZ | 7683423.75 | 27.7 |
| TGM3 | 7230481.46875 | 76.6 |
| PRDX1 | 7008305.90625 | 22.1 |
| HSP90B1 | 6934812.703125 | 92.4 |
| PCMTD1 | 6662231.5 | 40.7 |
| CAT | 6149919.875 | 59.7 |
| MCCC1 | 6149884 | 80.4 |
